# A mathematical model for simulation of cardiovascular, renal, and hormonal responses to burn injury and resuscitation

**DOI:** 10.3389/fphys.2024.1467351

**Published:** 2024-10-03

**Authors:** Ghazal ArabiDarrehDor, George C. Kramer, David M. Burmeister, Jose Salinas, Jin-Oh Hahn

**Affiliations:** ^1^ Mechanical Engineering, University of Maryland, College Park, MD, United States; ^2^ Anesthesiology, University of Texas Medical Branch, Galveston, TX, United States; ^3^ Department of Medicine, Uniformed Services University, Bethesda, MD, United States; ^4^ U. S. Army Institute of Surgical Research, San Antonio, TX, United States

**Keywords:** burn injury, burn resuscitation, mathematical model, kidney function, cardiovascular system, *in silico* testing, RAAS, virtual patient

## Abstract

**Introduction:**

Treating extensive burn injury requires an individually tailored resuscitation protocol that includes hourly-titrated intravenous fluid infusion to avert both hypovolemic shock and edema. Due to the complexity of burn pathophysiology and significant variability in treatment protocols, there is an ongoing effort to optimize burn resuscitation. The goal of this work is to contribute to this effort by developing a mathematical model of burn pathophysiology and resuscitation for *in silico* testing of burn resuscitation protocols and decision-support systems.

**Methods:**

In our previous work, we developed and validated a mathematical model consisting of volume kinetics, burn-induced perturbations, and kidney function. In this work, we expanded our previous mathematical model to incorporate novel mathematical models of cardiovascular system and hormonal system (renin-angiotensin-aldosterone (RAAS) system and antidiuretic hormone) which affect blood volume and pressure regulation. We also developed a detailed mathematical model of kidney function to regulate blood volume, pressure, and sodium levels, including components for glomerular filtration rate, reabsorption rates in nephron tubules, Tubuglomerular feedback, and myogenic mechanisms. We trained and validated the expanded mathematical model using experimental data from 15 pigs and 9 sheep with extensive burns to quantitatively evaluate its prediction accuracy for hematocrit, cardiac output, mean arterial pressure, central venous pressure, serum sodium levels, and urinary output. We then trained and tested the mathematical model using a clinical dataset of 233 human burn patients with demographic data and urinary output measurements.

**Results:**

The mathematical model could predict all tested variables very well, while internal variables and estimated parameters were consistent with the literature.

**Discussion:**

To the best of our knowledge, this is the first mathematical model of burn injury and resuscitation which is extensively validated to replicate actual burn patients. Hence, this *in silico* platform may complement large animal pre-clinical testing of burn resuscitation protocols. Beyond its primary purpose, the mathematical model can be used as a training tool for healthcare providers delivering insight into the pathophysiology of burn shock, and offering novel mathematical models of human physiology which can be independently used for other purposes and contexts.

## 1 Introduction

Patients with extensive burn injuries typically require substantial amounts of intravenous (IV) fluid to maintain vital organ perfusion and restore homeostasis. While many types of traumas require IV fluid replacement, fluid replacement treatment is uniquely challenging in the case of burn injury due to the multifactorial inflammation and endotheliopathy which results in a large amount of plasma shifting from the intravascular space into the tissues (Cartotto et al., 2022). Hence, a considerable fraction of the IV fluid given to replace the lost blood volume (BV) could likewise leak into the burnt and intact tissues, resulting in massive edema. To avoid both hypovolemia and hypervolemia as well as to minimize the risk of complications, e.g., hypovolemic shock, abdominal compartment syndrome, pulmonary edema, and organ failure, it is essential to optimize the dose of fluid given to a patient using clinical endpoints suited to guide the resuscitation based on patient response (i.e., precision medicine).

In burn centers, resuscitation often starts with an established burn resuscitation protocol such as the Parkland or modified Brooke formulas, which recommends an initial fluid dose based on weight (W) and total burned surface area (TBSA). The dose is then frequently titrated in an *ad hoc* fashion to clinical endpoints of choice. A commonly used endpoint is urinary output (UO), which is viewed as a proxy for intravascular BV. In this case, the goal is to maintain UO in the therapeutic target range of 30–50 mL/h or 0.5–1.0 mL/kg.hr (Schaefer and Nunez Lopez, 2023; Greenhalgh, 2010).

Regardless, existing burn resuscitation protocols exhibit large variability in treatment efficacy, due to many factors such as patient-to-patient variation in pre-existing comorbidities and in resuscitation response, incomplete and limited knowledge of burn pathophysiology, and challenges associated with the decision-making process involved in manual adjustment of fluid dose. Hence, the optimization and individualization of burn resuscitation protocols through the development of new decision-support algorithms and systems are an active area of research. This poses new challenges, since each protocol must be thoroughly evaluated before it can be clinically adopted. It is unethical to test a new treatment with unproven efficacy and safety profile in critically-ill burn patients. Additionally, large-scale pre-clinical tests on large mammals, such as sheep and pigs, likewise pose financial and ethical costs.

In this context, a credible and comprehensive mechanistic mathematical model of burn injury and resuscitation offers many benefits. Most relevantly, it can serve as a valuable platform for *in silico* evaluation of resuscitation protocols by virtue of its ability to provide insights into burn injury and resuscitation through the prediction of variables that are not measurable (Arabidarrehdor et al., 2021a). In addition, such a mechanistic mathematical model can be used as a training tool for healthcare professionals, which, given the complexity of burn resuscitation decision-making, is a notable advantage.

In our prior work, we developed a mathematical model of burn injury and resuscitation (Arabidarrehdor et al., 2021a; Arabidarrehdor et al., 2021b). The mathematical model consisted of three main components: (i) volume kinetics (VK), a mechanistic three-compartment mathematical model of water and albumin kinetics; (ii) a hybrid mechanistic phenomenological model of kidney function; and (iii) a phenomenological model of the disruptions inflicted on the body by burns and the ensuing inflammatory storm. We validated our mathematical model using data from sheep (N = 16) and humans (N = 233) with extensive burn injuries, which showed that it may predict VK and kidney function response to a range of burn injury severities and resuscitation fluid doses.

Although the predictions of the original mathematical model were consistent with the experimental data as well as the existing knowledge in the literature, there were opportunities to further improve the mathematical model especially in the context of enhancing physical transparency and clinical relevance. An aspect of particular interest was to expand the mathematical model so that it can embed the intricate interconnections between the regulation of BV, blood pressure (BP), and electrolyte balance in the body, given their role in maintaining adequate fluid perfusion.

In our prior work, we mainly focused on VK after burn injury. Yet, from a clinical standpoint, cardiovascular (CV) variables such as cardiac output (CO), mean arterial pressure (MAP), and central venous pressure (CVP) are of great importance. The primary objective of resuscitation in burn injury and many other forms of shock-inducing trauma is to restore end organ perfusion, which is often determined by CO, or a combination of vital signs including CO and MAP (Meng et al., 2015; Hasanin et al., 2017; Carr, 2012). Although UO is used to guide burn resuscitation in >94% of burn centers due to its convenience and non-invasiveness, its limitations in estimating tissue perfusion by itself are well-known, as indicated by a recent interest in combining hemodynamic monitoring and other endpoints with UO for optimal burn resuscitation (Paratz et al., 2014; Dries and Waxman, 1991; Caruso and Matthews, 2016). Considering that the ability to accurately predict CV variables (namely, CO, MAP, and CVP) in addition to UO and VK could be valuable to the clinical impact of a mathematical model intended for simulating burn injury and resuscitation, we expanded our prior mathematical model by incorporating CV physiology including autonomic nervous system as well as by integrating renin-angiotensin-aldosterone system (RAAS), which together influence short-term and long-term regulation of BV, electrolyte, and BP in the body. Note that expanding the ability of the mathematical model to simulate a more extensive set of variables also helps lift a few simplifying assumptions made in the original mathematical model, especially those associated with the kidney function. First, a mathematical model of RAAS enables a physiologically transparent mechanistic description of the sodium dynamics in the body. Second, a mathematical model of CV physiology enables the estimation of upstream and downstream renal arterial pressures. We take advantage of these opportunities by developing a more accurate, mechanistic mathematical model of the kidney function in this work.

We validated the mathematical model of burn injury and resuscitation extended as described above using two experimental datasets collected from 15 pigs and 9 sheep with extensive burn injuries that were resuscitated toward varying ends and, subsequently, a real clinical dataset of 233 patients with a wide range of burn severity and treatment outcomes. To the best of our knowledge, this is the first mechanistic mathematical model of burn injury and resuscitation which has been rigorously validated using diverse datasets.

This paper is organized as follows. In Section 2.1, we provide an overview of the mathematical model and the components therein. Section 2.2 explains the datasets as well as training and validation methods. Section 3 presents and discusses the results. Section 4 concludes the paper with a summary of contributions.

## 2 Material and methods

### 2.1 Mathematical model development

**FIGURE 1:**
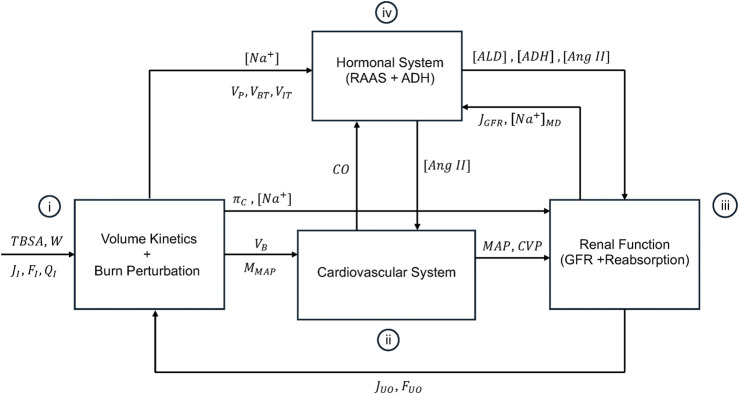
Schematic of the mathematical model of burn injury and resuscitation. It consists of (i): volume kinetics (VK) and burn-induced perturbation developed in our prior work, (ii), a mathematical model of CV system relevant to burn injury and resuscitation, (iii), a mathematical model of kidney function which can simulate glomerular filtration rate (GFR) and the reabsorption rates for water and sodium, and (iv) a mathematical model of hormonal systems including RAAS and antidiuretic hormone (ADH), which altogether modulate the regulation of water, sodium, and BP. The inputs to the mathematical model are TBSA, W, and fluid dose, all inputted into VK and the mathematical model of burn-induced perturbation.

Figure 1 shows the schematic of the mathematical model of burn injury and resuscitation. It consists of: (i) VK and burn-induced perturbations developed in our prior work, (ii) a mathematical model of CV system relevant to burn injury and resuscitation, (iii) a mathematical model of kidney function which can simulate glomerular filtration rate (GFR) as well as water and sodium reabsorption rates, and (iv) a mathematical model of hormonal systems including RAAS and antidiuretic hormone (ADH), which altogether modulate the regulation of water, sodium, and BP. Conceptual explanations on these components and how they are interconnected with each other are provided in this paper, while all the mathematical details (namely, the governing equations) are provided in Supplementary Material. All the variables and abbreviations are defined in Supplementary Table S1 in Supplementary Material. All the parameter values are summarized in Supplementary Table S2 in Supplementary Material.

#### 2.1.1 Volume kinetics and burn-induced perturbations

We used a mathematical model of VK and burn-induced perturbations developed in our prior work (Arabidarrehdor et al., 2021a; Arabidarrehdor et al., 2021b), which includes three compartments: the intravascular space (i.e., plasma), burned tissues (skin), and intact tissues (including intact skin, muscle tissues, and the rest of interstitial space). The mathematical model of VK and perturbation encompasses 7 states in the form of ordinary differential equations (ODEs) which fully describe the distribution of the following elements in the body: plasma water volume ( $V_{P}$ ), plasma albumin content ( $A_{P}$ ), water volumes in burnt and intact tissues ( $V_{BT}$ and $V_{IT}$ ), albumin contents in burnt and intact tissues ( $A_{BT}$ and $A_{IT}$ ), and extracellular sodium content ( $Na_{ECF}^{+}$ ). The initial values of all these states are determined by W and TBSA.

$V_{P}$ , $V_{BT}$ , and $V_{IT}$ are primarily modulated by capillary filtration of fluid and lymphatic drainage. Capillary filtration perfuses the interstitium at a rate determined by membrane properties and the pressure gradients between plasma and tissue, which we modeled using the Starling equations. We represented the hydrostatic pressure-volume relationships in the intravascular compartment and the interstitial tissues as linear and nonlinear mechanistic mathematical models, respectively. In addition, we likewise used linear mathematical models to estimate the colloid oncotic pressure from the albumin concentration in each compartment. The lymphatic network drains the excess fluid from the tissues and returns it back to plasma to maintain homeostasis. We modeled the lymphatic flow rate as a sigmoid function of the tissue hydrostatic pressure. $V_{P}$ is also dependent on UO ( $J_{UO}$ ), which is predicted by a mathematical model of kidney function (see Section 2.1.3), and the IV fluid dose ( $J_{I}$ ). $V_{BT}$ and $V_{IT}$ are not directly affected by fluid dose and UO, but are affected by dermal fluid loss in the form of evaporation from the skin and exudation from the wound, both modeled using empirical equations.

$A_{P}$ , $A_{BT}$ , and $A_{IT}$ are mainly determined by the capillary filtration of albumin, estimated using the coupled diffusion-convection equation (Chapple et al., 1993; Bresler and Groome, 1981) and the free transport of albumin in the lymphatic flow. $A_{P}$ is additionally affected by the albumin introduced via IV fluid ( $Q_{I}$ , zero in case of the lactate ringers (LR)), and $A_{BT}$ by the protein denaturation in the burnt tissue as a result of heat.

Since the fluid exchange pertaining to both capillary filtration and lymphatic flow are isotonic, $Na_{ECF}^{+}$ is solely influenced by the sodium gain through fluid infusion ( $F_{I}$ ) and sodium loss through UO ( $F_{UO}$ ).

Extensive burns introduce perturbations in burnt tissues (including capillary destruction, protein denaturation, negative pressure in burnt tissue, and dermal fluid loss) as well as in systemic circulation (including increased capillary pore size, vasodilation, and systemic vasoconstriction). These disruptions lead to a significant increase in capillary filtration, which can cause hypovolemia and edema. We described these perturbations by an array of phenomenological models whose effects last transiently and disappear. The severity and time courses of these disruptions are calibrated based on individual responses, by specifying subject-specific parameters pertaining to each perturbation. In addition to the intensity aspect of the injury severity (which is represented by the perturbation parameters), injury severity is also represented by specifying the size of the burned tissues according to the subject’s W and TBSA. This approach captures the “extent” aspect of the injury severity. Full details and equations on the mathematical model of VK and burn-induced perturbations are described in our prior work and in Section S1 in Supplementary Material.

In sum, the mathematical model of VK and burn-induced perturbations receives as inputs (i) W, TBSA, baseline hematocrit (HCT), and fluid dose from the dataset; and (ii) UO ( $J_{UO}$ and ( ${\left[Na^{+}\right]}_{UO}$ ) predicted by the mathematical model of kidney function (Figure 1). Then, it furnishes (i) $V_{P}$ , $V_{BT}$ , $V_{IT}$ , and plasma sodium concentration ( $\left[Na^{+}\right]$ ) to the mathematical model of hormonal system; (ii) BV ( $V_{B}$ ) and the magnitude of vasoconstriction ( $M_{MAP}$ ) to the mathematical model of CV system; and (iii) ( $\left[Na^{+}\right]$ and the capillary colloid oncotic pressure ( $\pi_{C}$ ) to the mathematical model of kidney function (Figure 1).

#### 2.1.2 Cardiovascular physiology

Mathematical modeling of the CV system has been an attractive research topic for a long time due to its incredible complexity and multiscale nature. Among the myriad mechanistic and data-driven mathematical models in the literature (Tivay et al., 2020; Quarteroni et al., 2017; Kappel and Peer, 1993; Abdolrazaghi et al., 2010; D’Orsi et al., 2021; Kislova et al., 2006; Karavaev et al., 2016; Bozkurtid, 2019), we adopted and extended the mathematical model of CV system proposed by Uttamsingh et al. (1985), primarily motivated by its simplicity and its ability to predict long-term fluctuations in the CV system.

This mathematical model is principally developed based on Guyton’s famous CO-venous return (VR) curve and empirical equations derived from clinical studies. The Guyton’s CO-VR curve has no closed-form solutions and requires an iterative numerical approach for its solution which is inefficient in long-term simulations. In addition, the empirical equations are built upon clinical data predating 1985. Thus, some of them may be outdated relative to the contemporary knowledge in the literature. Further, the empirical equations may not be universally valid in the three species used in this work. Hence, we enhanced the robustness and adaptability of this mathematical model according to our context of use, by (i) numerically solving the CO-VR curve using simplified assumptions drawn from the existing literature, (ii) incorporating recent experimental findings to update the empirical equations in the mathematical model, and (iii) tailoring the equations to enable the selection of diverse initial conditions and responses to accommodate the heterogeneity in our datasets.

**FIGURE 2:**
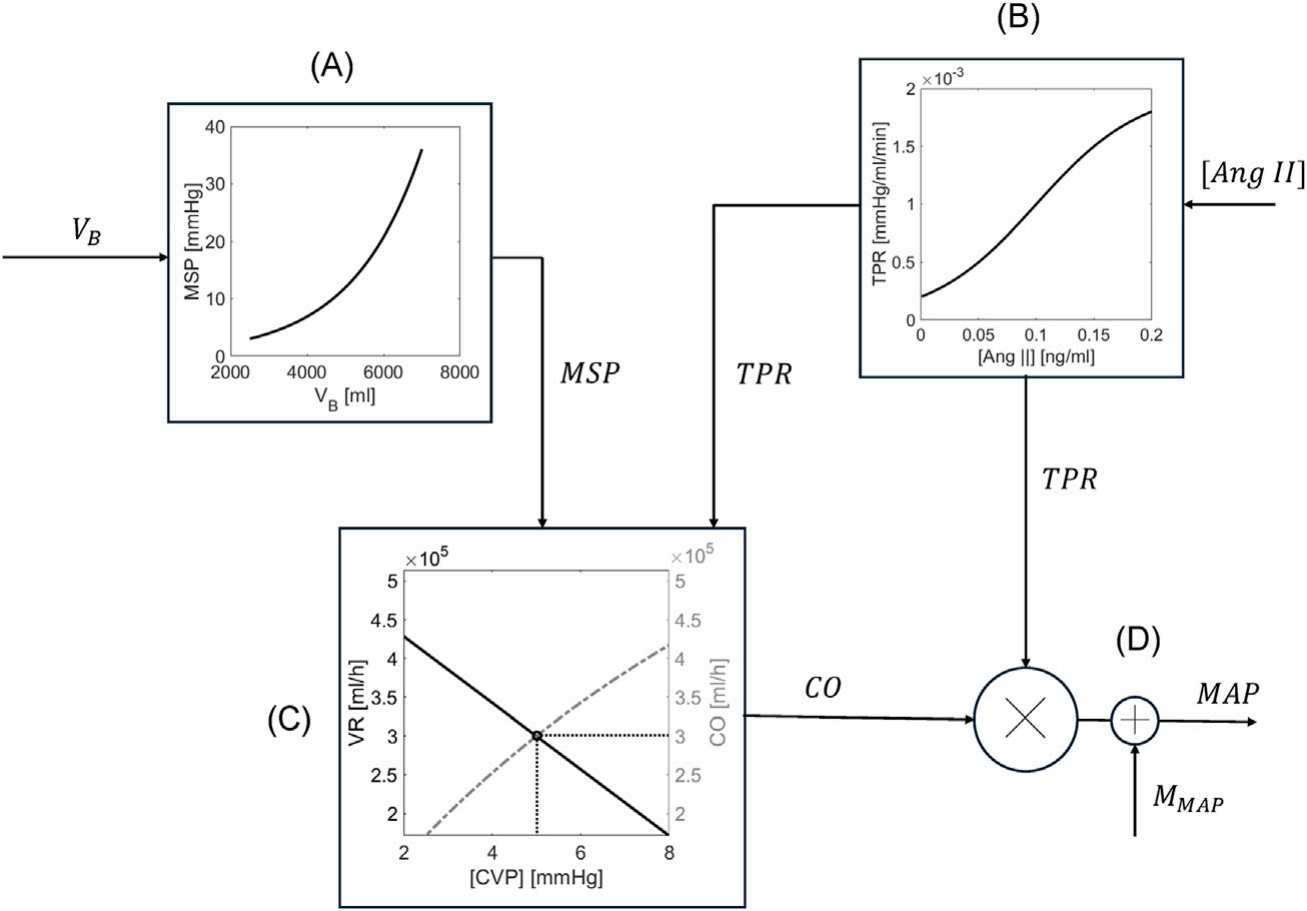
Schematic of the mathematical model of cardiovascular system. **(A)** MSP is estimated from $V_{B}$ furnished by the mathematical model of VK using an exponential relationship. **(B)** TPR is estimated from $\left[Ang\;II\right]$ furnished by the mathematical model of hormonal system using a sigmoidal relationship. **(C)** CO is estimated from VR and CVP using a combination of the Frank-Starling law and Guyton’s CO-VR curves. **(D)** MAP is calculated from CO, TPR, and $M_{MAP}$ furnished by the mathematical model of burn-induced perturbations.

A decrease in BV lowers the pressure within the veins, i.e., the mean systemic pressure (MSP), which reduces the upstream pressure determining VR and subsequently diminishes CO and MAP. The RAAS responds to the decline in MAP by releasing renin and increasing plasma angiotensin II (Ang II) concentration ( $\left[Ang\;II\right]$ ), which elicits vasoconstriction. The constricted arterioles raise total peripheral resistance (TPR), which ultimately increases MAP and CO until normal conditions are restored. We have modeled this sequence of events and the complex relationships between these CV variables using four components illustrated in Figure 2. The component (A) receives $V_{B}$ as an input from the mathematical model of VK and estimates MSP using an exponential function in agreement with a recent study (W et al., 1974). The component (B) receives $\left[Ang\;II\right]$ as an input from the mathematical model of hormonal system and estimates TPR using a sigmoidal relationship. The component (C) dictates how VR changes as a linear function of CVP, with the slope of the linear function determined by MSP and TPR received as inputs from (A) and (B). In addition, the component (C) represents CO as a dose-response function of CVP. Here, we utilize the Frank-Starling law to find the true value of CO as the intersection point of CO curve and VR curve (known as circulatory equilibrium). Finally, in the component (D), MAP is calculated using CO, TPR, and $M_{MAP}$ furnished by the mathematical model of burn-induced perturbations. Full details and equations on the mathematical model of CV system are summarized in Section S2 in Supplementary Material.

In sum, the mathematical model of CV system receives as inputs (i) $V_{B}$ from the mathematical model of VK, (ii) $M_{MAP}$ from the mathematical model of burn-induced perturbations, and (iii) $\left[Ang\;II\right]$ from the mathematical model of hormonal system. Then, it furnishes (i) CO to the mathematical model of hormonal system and (ii) MAP and CVP to the mathematical model of kidney function (Figure 1).

#### 2.1.3 Kidney function

**FIGURE 3:**
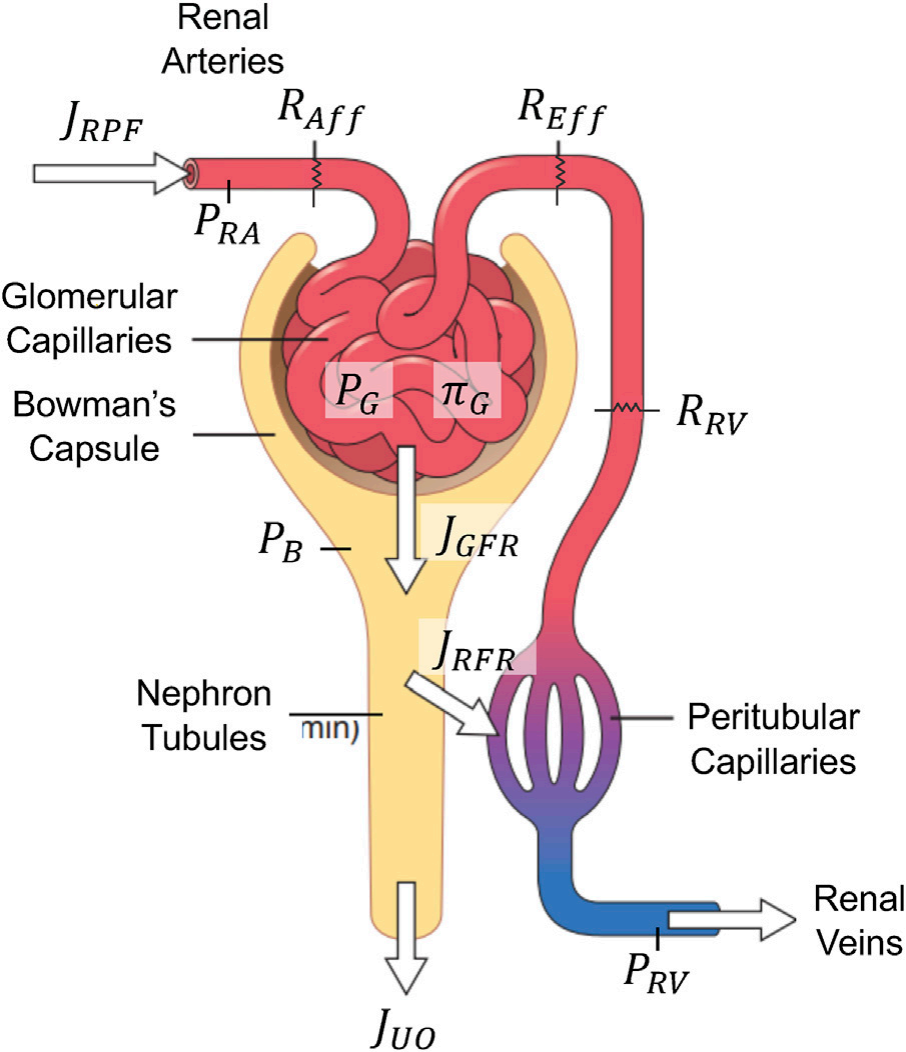
Schematic of the mathematical model of glomerular filtration and renal plasma flow regulation. Adapted with permission of Elsevier Science & Technology Journals, from Guyton and Hall, 2011a; permission conveyed through Copyright Clearance Center, Inc.

Figure 3 shows a high-level schematic of the kidney function relevant to GFR and renal plasma flow (RPF) regulation. Plasma flows into the kidneys through renal arteries, which branch into capillaries running along millions of parallel nephrons. In every nephron, fluid passes through afferent arterioles into the glomerular space, where approximately 20% of the fluid (called the filtrate) is filtered into Bowman’s capsule. The remaining 80% flows through efferent arterioles, peritubular capillaries, and eventually, renal venules. Along this path, fluid is joined by the majority of the filtrate as it is reabsorbed from the nephron tubules back into circulation. Eventually, approximately just 1% of RPF is excreted as UO under normal conditions. RPF, GFR, and UO are meticulously regulated by a complex set of interconnected mechanisms in the body, including components from VK, CV, and hormonal systems. To cast this sophisticated kidney function into a simple mathematical model, we first assumed that all nephrons are homogeneous in both characteristics and resistances. Then, we developed a mathematical model to predict the collective flows in the kidneys to represent the dynamics of the entire kidneys.

##### 2.1.3.1 Glomerular filtration rate and renal plasma flow regulation

RPF rate ( $J_{RPF}$ ) is calculated using the Poiseuille’s law, where it is directly proportional to the pressure drop across kidney and inversely proportional to total renal resistance. Renal arterial pressure ( $P_{RA}$ ) and renal venous pressure are linear functions of MAP and CVP, respectively, which are furnished by the mathematical model of CV system (Figure 1). GFR ( $J_{GFR}$ ) is a fast and highly pressurized capillary filtration and is calculated from Starling forces formed by hydrostatic ( $P_{G}$ ) and colloid oncotic ( $\pi_{G}$ ) pressures in the glomerulus. Higher input flow and larger downstream resistance can increase hydrostatic pressure. Hence, $P_{G}$ is assumed to be proportional to $J_{RPF}$ as well as to the sum of renal efferent resistance ( $R_{Eff}$ ) and renal venous resistance ( $R_{RV}$ ). $\pi_{G}$ is not uniform in the glomerulus since albumin concentration is continuously increased by filtration of water into the Bowman’s capsule. This phenomenon makes $\pi_{G}$ dependent on GFR, as a larger GFR means a faster rate of increase in $\pi_{G}$ . Despite the complexity of the kidney function dynamics, if we assume that the equilibrium among the elements in the Starling forces is eventually achieved somewhere along the glomerular capillaries (Deen et al., 1972; Brenner et al., 1972), we can estimate $\pi_{G}$ as a linear function of $P_{G}$ , $P_{B}$ , and $\pi_{C}$ which is furnished by the mathematical model of VK.

To maintain kidney function, the kidneys have two intrinsic mechanisms that modulate $J_{RPF}$ and $J_{GFR}$ by adjusting the renal resistances: myogenic mechanism (MM) and tubuglomerular feedback (TGF). In MM, the smooth muscle cells in the afferent arterioles respond to changes in $P_{RA}$ by adjusting the diameter of the arterioles, and consequently, the afferent resistance ( $R_{Aff}$ ). TGF responds to the variations in sodium concentration at macula densa (MD) cells located in the distal tubules. Simply put, higher $J_{GFR}$ delivers more sodium to MD, increasing its concentration therein ( ${\left[Na^{+}\right]}_{MD}$ ). MD cells sense this elevation and send commands to constrict afferent arterioles and lower input flow rates. Thus, the part of $R_{Aff}$ controlled by TGF ( $R_{TGF}$ ) is determined by a sigmoid function of ${\left[Na^{+}\right]}_{MD}$ . In addition, TGF has functions to regulate MAP and $J_{GFR}$ in a longer-term fashion, where MD cells inhibit the release of renin in response to an increase in ${\left[Na^{+}\right]}_{MD}$ . In addition, through a mechanism which will be explained in Section 2.1.4.1, Ang II release rate is also reduced, which dilates arterioles and reduces MAP. The dilation of efferent arterioles in particular, reduces $R_{Eff}$ , which in turn reduces $J_{GFR}$ .

In sum, the mathematical model of GFR and RPF regulation receives as inputs (i) MAP and CVP from the mathematical model of CV system, $\pi_{C}$ from the mathematical model of VK, ${\left[Na^{+}\right]}_{MD}$ from the mathematical model of kidney reabsorption (to be explained in Section 2.1.3.2), and plasma Ang II concentration from the mathematical model of hormonal system (RAAS). Then, it furnishes $J_{GFR}$ and ${\left[Na^{+}\right]}_{MD}$ to the mathematical model of hormonal system. Full details and equations on the mathematical model of GFR and RPF regulation are summarized in Section S3.1 in Supplementary Material.

##### 2.1.3.2 Reabsorption of water and sodium

**FIGURE 4:**
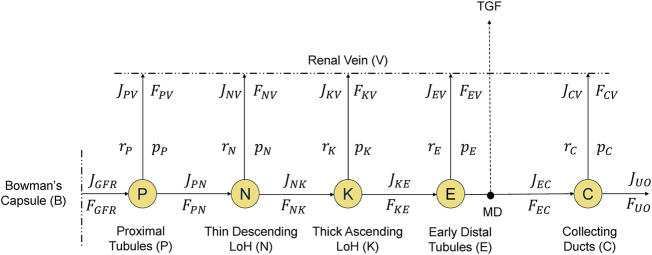
A node-schematic of the mathematical model of renal reabsorption. Water and sodium flow into Bowman’s capsule and then into nephron tubules, where they are reabsorbed back into the circulation through renal vein at different rates along the way, while the remaining filtrate is excreted as urinary output (UO). Tubuloglomerular feedback (TGF) is activated by sodium concentration at macula densa (MD). $J_{XY}$ and $F_{XY}$ : water flow and sodium flow from node X to node Y. $J_{XV}$ and $F_{XV}$ : water flow and sodium flow from node X to the renal vein (V), i.e., back to the circulation (reabsorption). $r_{X}$ and $p_{X}$ : reabsorption fraction of water and sodium at node X.

Figure 4 is a node schematic of the mathematical model of renal reabsorption, which unravels the convoluted structure of nephrons. Water and sodium are filtered from glomerular capillaries into Bowman’s capsule at a rate of $J_{GFR}$ . Then, the filtrate flows through different segments of nephron (nodes in Figure 4), i.e., proximal tubules, the loop of Henle and its two limbs, and distal and collecting tubules, while it is reabsorbed back into the circulation along the way. The remaining fluid empties through collecting ducts into the ureter and the bladder, where it is excreted as UO. The characteristics of reabsorption of water and sodium are entirely different in these segments. Hence, it is imperative to capture these differences in order to correctly implement TGF and hormonal functions.

In this schematic, $J_{XY}$ and $F_{XY}$ represent water flow and sodium flow from node X to node Y, while $J_{XV}$ and $F_{XV}$ represent water flow and sodium flow from node X to the renal vein (V), i.e., back to the circulation (reabsorption). At each node, $r_{X}$ and $p_{X}$ represent the fraction of flow reabsorbed into the renal vein for water and sodium, respectively.

At the proximal tubules (“P” in Figure 4), water and sodium are reabsorbed together at the same rate as regulated by the glomerulotubular balance and plasma aldosterone concentration ( $\left[ALD\right]$ ). A dose-response relationship describes the relationship between $r_{P}$ and $\left[ALD\right]$ (Good, 2007; Salyer et al., 2013; Guyton, 1975), which acts to maintain the reabsorption fraction at its baseline level of 65%–75% (Uttamsingh et al., 1985; Czerwin et al., 2021; Moss and Thomas, 2014). The sodium reabsorption rate in the proximal tubules is also influenced by Ang II. Given that Ang II indirectly affects sodium reabsorption by enhancing aldosterone production (see Section 2.1.4.1), we chose not to model the direct effects of Ang II on the sodium reabsorption rates at the proximal tubule in order to promote the simplicity of the mathematical model.

The thin descending limb of Henle (“N” in Figure 4) is impermeable to sodium. As a result, only water is reabsorbed passively: slower flow ( $J_{PN}$ ) allows for increased reabsorption ( $r_{N}$ ). This results in an inverse relationship between flow rate and reabsorption fraction, which is one of the major players in pressure diuresis. We captured this phenomenon by an inverse-sigmoidal function between $r_{N}$ and $J_{PN}$ . The thick ascending limb of Henle (“K” in Figure 4), on the other hand, is impermeable to water. As a result, it actively reabsorbs approximately 60% of the sodium inflow ( $F_{NK}$ ).

In the early distal tubules (“E” in Figure 4), we still have water impermeability. However, its sodium reabsorption ( $p_{E}$ ) is passive, having an inverse relationship with sodium flow rate ( $F_{KE}$ ). We used an inverse-sigmoidal function of the sodium flow, similar to the thin descending limb of Henle, to describe the relationship between $p_{E}$ and $F_{KE}$ . Since there is no more reabsorption between the early distal tubules and MD, sodium concentration at this node is equal to ${\left[Na^{+}\right]}_{MD}$ .

Finally, at the collecting ducts (“C” in Figure 4), the reabsorption of water and sodium is regulated by hormones, i.e., ADH for water and aldosterone for sodium. We have used dose-response curves to describe the relationship between $r_{C}$ and plasma ADH ( $\left[ADH\right]$ ) as well as $p_{C}$ and $\left[ALD\right]$ . Any unabsorbed fluid at this stage proceeds to the bladder for excretion as UO.

In sum, the mathematical model of renal reabsorption receives as inputs (i) $J_{GFR}$ from the mathematical model of glomerular filtration, (ii) $\left[Na^{+}\right]$ from the mathematical model of VK (to estimate $F_{GFR}$ ), and (iii) plasma concentrations of ADH and aldosterone from the mathematical model of hormonal system. Then, it furnishes (i) the fluctuations in ${\left[Na^{+}\right]}_{MD}$ to the mathematical models of glomerular filtration (thereby modulating both afferent and efferent renal resistances through TGF) and hormonal system (RAAS in particular) and (ii) $J_{UO}$ and urinary sodium concentration ${\left[Na^{+}\right]}_{UO}$ to the mathematical model of VK (Figure 1). Our modeling of the reabsorption function incorporates meticulous constraints and parameter bounds to ensure the credibility of predictions while facilitating individualization. Full details and equations on the mathematical model of renal reabsorption are summarized in Section S3.2 in Supplementary Material.

#### 2.1.4 Hormonal system

##### 2.1.4.1 Renin-angiotensin-aldosterone system

RAAS is a multi-factor mechanism for long-term regulation of MAP, BV, and electrolytes. Renin is part of TGF. When MD cells send signals to change the resistance of renal afferent arterioles in response to a change in MD sodium load, they also send signals to change the renin release rate. Specifically, a drop in BV and MAP as a result of severe burn will reduce RPF ( $\left.J_{RPF}\right)$ , GFR ( $J_{GFR}$ ), and subsequently, ${\left[Na^{+}\right]}_{MD}$ . There is an inverse relationship between renin release rate and ${\left[Na^{+}\right]}_{MD}$ [149]. Hence, a drop in GFR eventually increases renin release rate. In the mathematical model of RAAS system, the release of renin is linearly proportional to the fractional variations in ${\left[Na^{+}\right]}_{MD}$ , with its clearance facilitated through transit in both the liver and the kidneys.

Renin converts its substrate, angiotensinogen, to angiotensin I, a precursor for the vasoconstrictor hormone angiotensin II (Ang II) (Guyton, 1975). Ang II constricts arterioles and increases TPR, which ultimately increases MAP and GFR**.** In the mathematical model, the release of Ang II is linearly associated with the fractional changes in plasma renin concentration (with a delay), and its clearance occurs through hepatic circulation.

Finally, Ang II increases aldosterone release rate, which leads to sodium and water retention by the kidneys, thereby increasing BV and partially compensating for blood loss (Guyton, 1975). Aldosterone secretion is stimulated by two distinct sources: (i) it has a negative linear relationship with $\left[Na^{+}\right]$ to reabsorb more sodium in the collecting ducts when $\left[Na^{+}\right]$ decreases, and (ii) it has a positive relationship with Ang II. We modeled these relationships using a dose-response curve based on experiments conducted by Uttamsingh et al. (1985); Blair-West et al. (1962), where total aldosterone release is calculated as an exponential function of the weighted sum of the two sources. Like renin, it is eliminated by both hepatic and renal blood flows. The secretion rate of aldosterone is also influenced by serum potassium levels, which we assume to be constant in this study for the sake of simplicity.

In sum, the mathematical model of RAAS system receives as inputs (i) ${\left[Na^{+}\right]}_{MD}$ from the mathematical model of renal reabsorption and (ii) $\left[Na^{+}\right]$ from the mathematical model of VK. Then, it furnishes (i) Ang II to the mathematical model of CV system and (ii) $\left[Ang\;II\right]$ and $\left[ALD\right]$ to the mathematical model of kidney function. Full details and equations on the mathematical model of RAAS are summarized in Section S4.1 in Supplementary Material.

##### 2.1.4.2 Antidiuretic hormone

The ADH content is modulated by signals from baroreceptor (inversely related to the changes in $V_{P}$ ) and osmoreceptor (directly related to the changes in $\left[Na^{+}\right]$ ). ADH affects the rate at which pure water is reabsorbed at the collecting ducts (Guyton and Hall, 2011b; Voets and Maas, 2018) (see Eq. (S79) in Section S3.2 in Supplementary Material). Specifically, when $V_{P}$ decreases, more ADH is released to increase the pure water reabsorption and retain more water in the body to compensate for the loss in plasma volume. An increase in plasma sodium concentration has the same effect, although with a larger sensitivity (Guyton and Hall, 2011b). We expressed the dynamics of the ADH content by a phenomenological model, where its secretion rate is an exponential function of the weighted sum of fractional changes in $V_{P}$ and $\left[Na^{+}\right]$ , and its elimination is facilitated by its passage through the liver and the kidneys (Guyton and Hall, 2011b; Voets and Maas, 2018; Heller and Zaidi, 1957). In sum, the mathematical model of ADH receives as inputs $V_{P}$ and $\left[Na^{+}\right]$ from the mathematical model of VK. Then, it furnishes $\left[ADH\right]$ to the mathematical model of renal reabsorption. Full details and equations on the mathematical model of ADH are summarized in Section S4.2 in Supplementary Material.

### 2.2 Mathematical model training and testing

#### 2.2.1 Experimental and clinical datasets

The data we used to train and validate our mathematical model in this work comes from 3 species: pigs, sheep, and humans. Pigs and sheep are widely used as a replacement for human subjects in pre-clinical and exploratory experiments by virtue of their physiological and anatomical similarity to humans (Walters and Prather, 2013; Banstola and Reynolds, 2022). Utilizing both experimental and clinical data allows for robust validation of the mathematical model while granting access to physiological variables typically unmeasured in patients due to ethical constraints. Table 1 summarizes the datasets. Details follow.

**TABLE 1:** Experimental and clinical datasets for mathematical model training and testing.

	Pigs	Sheep	Humans	
			Training	Testing
Subject Number	15	8	120	133
TBSA [%]	40	40	42 ± 18	38 ± 18
W [kg]	31.7 ± 4.2	40	85 ± 18	86 ± 22
Resuscitation Paradigm	[P1] No IV fluids [P2] UO: 1–1.5 mL/kg/h [P3] Over-resuscitation	UO: 1–2 mL/kg/h	UO: 30–50 mL/h	
Measurements	UO, MAP, CO, CVP $\left[Na^{+}\right]$	HCT MAP, CO, CVP UO	UO	

##### 2.2.1.1 Pigs

We used a subset of data from a study where 21 female Yorkshire swine were subject to 40% TBSA and randomly assigned to each of the following resuscitation paradigms: (i) Paradigm 1 (P1): no IV fluids to under-resuscitate the animals, (ii) Paradigm 2 (P2): IV Lactated Ringers (LR) guided by UO according to the Burn Navigator™ to maintain a target UO of 1–1.5 mL/kg, or (iii) Paradigm 3 (P3): a high rate of ≥500 mL/h IV LR throughout the protocol to deliberately over-resuscitate the animals. Details of the protocol can be found in our previously published work (ArabiDarrehDor et al., 2022; Kao et al., 2024). Animals were monitored for 24 h post-injury, and had hourly measurements of IV fluid rate and UO, and measurements of HCT, MAP, CO, CVP, and $\left[Na^{+}\right]$ at hours 0, 1, 2, 3, 5, 9, 12, 18 and 24. We excluded HCT from our anlysis because the animals were not splenectomized, since pigs have a contractile spleen and is able to “autotransfuse”. We used 15 animals (5 from each paradigm) to train and internally validate the mathematical model.

This dataset provided us with two unique advantages which we could not have achieved with the other two datasets: (i) the $\left[Na^{+}\right]$ measurements presented an opportunity to test the mathematical model of sodium dynamics; and (ii) the diversity in the resuscitation protocols allowed us to test the ability of the mathematical model in predicting the outcomes of highly diverse treatment scenarios, ranging from extreme under-resuscitation to over-resuscitation.

##### 2.2.1.2 Sheep

Experimental dataset associated with the sheep came from a prior work (Elgjo et al., 2000), where adult sheep (N = 8) with the median weight of 40 kg were induced with full-thickness burn injury of 40% TBSA. Burn resuscitation by LR was initiated 1 h post-burn and continued for 48 h. Resuscitation was performed to maintain a target UO of 1–2 mL/kg/h, which is considered normal in sheep. Key measurements in the dataset used in this work include hourly records of fluid infusion and UO, and more sparse measurements of HCT, CVP, MAP, and CO.

##### 2.2.1.3 Humans

To develop and validate our human mathematical model, we integrated data from two clinical datasets described in a previous study (Arabidarrehdor et al., 2021a). The first dataset involved 207 burn patients treated with the Burn Navigator™ in a burn ICU, aiming for a target UO of 30–50 mL/h (Salinas et al., 2012; Salinas et al., 2011). The second dataset included 53 burn patients, with 29 under Burn Navigator™ treatment and 24 following conventional protocols. Hourly data on UO, LR dose, demographics (age, weight, and gender for the first dataset), TBSA, and the time of arrival were collected. The patients in the collective dataset had an average age of 47 ± 18 years, weight of 87 ± 22 kg, and TBSA of 40% ± 18%. The overall mortality rate was 30%. In the first source, 77% of the patients were male, and 11% had inhalation injuries. It is noteworthy that care providers were at liberty of overriding Burn Navigator™ recommendations at any time.

After excluding 27 subjects with <10 UO recordings, we randomly divided the dataset into a training group (N = 120) for internal validation and a test group (N = 113) to externally validate the optimized mathematical model with reduced bias. Demographic and injury severity comparisons between training and test groups showed comparable values (age: 45 ± 19 years vs. 49 ± 18 years; weight: 85 ± 18 kg vs. 86 ± 22 kg; TBSA: 41.5% ± 17.6% vs. 38 ± 18 in training and test datasets, respectively).

#### 2.2.2 Verification method

While in our dataset we have several measurements relevant to validation of the mathematical models of VK and CV system, quantitative validation of the kidney function is limited to UO and $\left[Na^{+}\right]$ . To enhance the reliability of the mathematical model of renal function, we simulated the mathematical models of renal function and RAAS with typical human parameter values from the literature across a range of renal arterial pressures and investigated the predictions. Then, we investigated if the internal regulatory mechanisms in the kidneys, including myogenic mechanism, TGF, and glomerulotubular balance, are plausibly implemented.

#### 2.2.3 Validation method

The mathematical model has 98 parameters in total, among which 12 can be found using constraints such as assumptions of steady-state before injury, or directly from the datasets. To determine the remaining 86 parameters, we first categorized them into subject-invariant and subject-specific parameters. Subject-specific (SS) parameters included (i) those whose values are expected to exhibit large inter-individual variability; (ii) those whose values have rarely been reported in the existing literature, or (iii) those which are associated with phenomenological components in the mathematical model. This resulted in a set of 46 SS parameters. In the case of the clinical dataset (Section 2.2.1.3) where we have limited data, we further reduced the number of SS parameters as explained further below. The remaining 40 parameters are assumed to be subject-invariant and were taken from literature (Supplementary Table S2).

For each subject within our three species, we determined the SS parameter values by concurrently minimizing the difference between all the measured physiological variables (listed in Table 1) and their counterparts predicted by the mathematical model. This was achieved by minimizing the following cost function (Arabidarrehdor et al., 2021a; Tivay et al., 2019):

$$\theta_{i}=\arg\min_{\theta }{\overset{˘}{J}}_{i}\left(\theta \right)=\arg\min_{\theta }\sqrt{\sum_{j=1}^{M_{i}}{\left(\sum_{k=1}^{D_{ij}}\frac{\left|y_{ij}^{d}\left(t_{k}\right)-y_{ij}\left(t_{k},\theta \right)\right|}{Y_{ij}}\;\right)}^{2}}$$ (1)

Where $\theta_{i}$ is the vector of subject-specific parameters estimated for subject i , $M_{i}$ is the number of total physiological variables measured in subject i during the experiment, $D_{ij}$ is the number of measurements associated with the physiological variable j , $y_{ij}^{d}\left(t_{k}\right)$ is the value of the physiological variable j associated with the subject i measured at time $t_{k}$ , $y_{ij}\left(t_{k},\theta \right)$ is the value of the same physiological variable at time $t_{k}$ predicted by the mathematical model equipped with θ , and $Y_{ij}$ is the normalization factor for the physiological variable j . We used the “globalsearch” command in MATLAB in conjunction with the “fmincon” command to robustly estimate the parameter values. In addition, we enforced tight parameter bounds as constraints in Equation 1 to effectively guide the solution into a mechanistically plausible parameter space.

To estimate 46 SS parameters for the pig subjects, we fit the mathematical model predictions to UO, MAP, CO, CVP, and $\left[Na^{+}\right]$ by minimizing the objective function in Equation 1 ( $M_{i}$ = 5). On the average, each pig subject had 94 available data points to estimate the 46 unknown parameters. Subject W, TBSA, baseline measurements of HCT, and hourly infusion rates were utilized from the dataset as inputs to conduct simulations.

For sheep subjects, we used UO, HCT, CVP, MAP, and CO ( $M_{i}$ = 5). It is worth noting that since sheep subjects had reliable baseline measurements, the number of subject-specific parameters was reduced to 44: we did not have to estimate the baseline CO and MAP. On the average, every sheep subject had 112 available datapoints to identify the 44 unknown parameters. Subject W, TBSA, baseline measurements of HCT, CO, and MAP, and hourly infusion rates were utilized from the dataset as inputs to conduct simulations.

For humans, the training set of 120 subjects was used to estimate unknown parameters by fitting the mathematical model to UO ( $M_{i}$ = 1). Since the ratio of the number of unknown parameters to data points was large (46–23 ± 2), we performed sensitivity analysis using a regularized population-average mathematical model [similar to our previous work (Arabidarrehdor et al., 2021a; Tivay et al., 2019)] to determine non-sensitive parameters and fixed them to population-average values, thereby preventing overfitting. Then, we externally validated the mathematical model with smaller number of SS parameters based on the test set subjects. Patient W, TBSA, and hourly infusion rates were utilized from the dataset as inputs to conduct simulations.

## 3 Results and discussion

### 3.1 Verification

**FIGURE 5:**
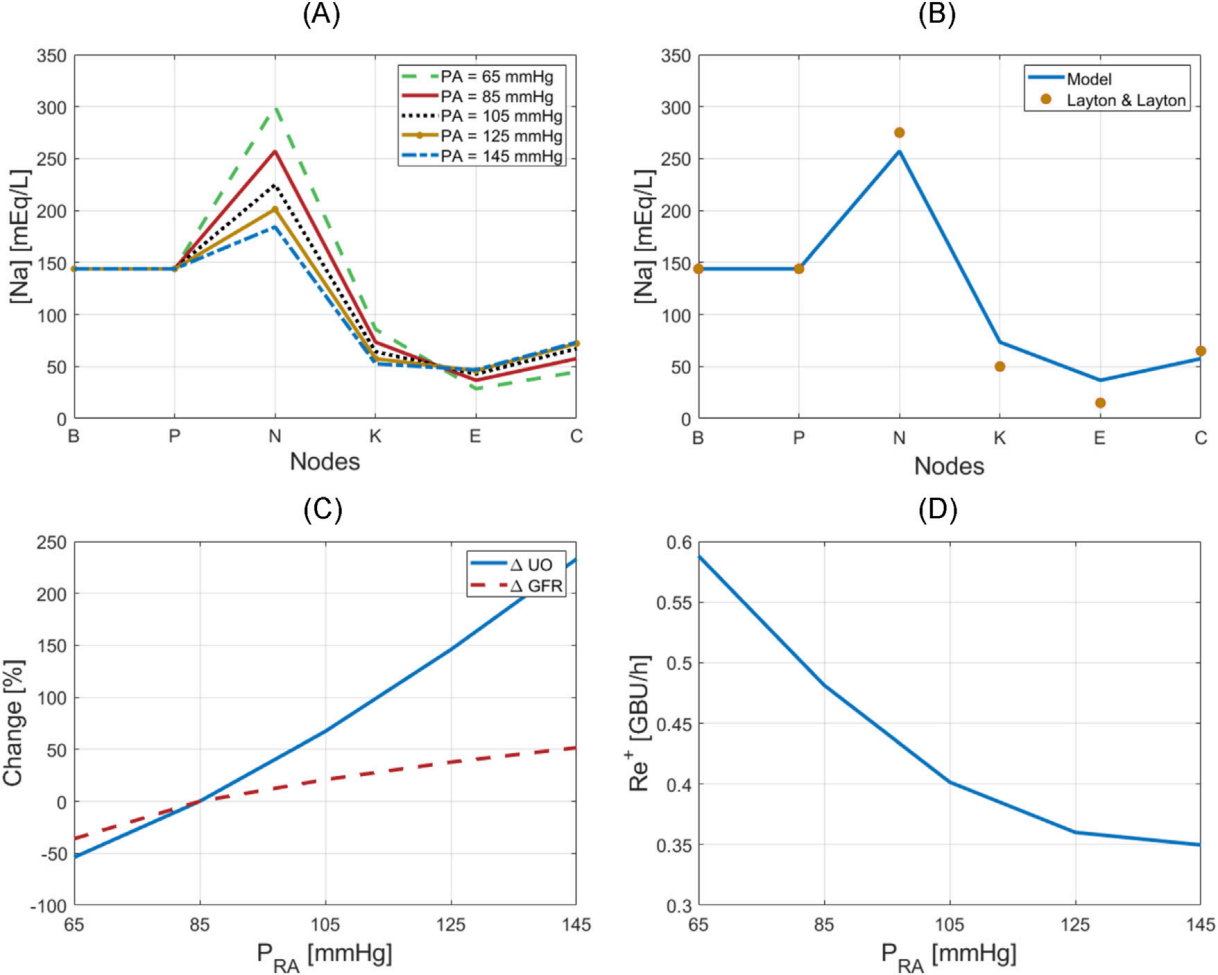
Verification of kidney function regulatory mechanisms. **(A)** Sodium concentration at nodes of the nephrons with respect to renal arterial pressure. **(B)** Nominal sodium concentration at nodes of the nephrons in comparison to simulations in Layton and Layton (Layton and Layton, 2019). **(C)** Variation in UO and GFR with respect to renal arterial pressure. **(D)** Renin release rate with respect to renal arterial pressure.

Figure 5 shows the results for the verification analysis of the kidney function regulatory mechanisms. Figure 5A shows how the sodium concentrations at different nodes in the nephrons vary when the renal arterial pressure deviates from a baseline value of 85 mmHg. The mathematical model predicted that sodium concentration at node P remains the same as plasma sodium concentration at node B across all renal arterial pressure levels, which is plausible because sodium and water are reabsorbed together at node B (see Section 2.1.3.2). At node N, the mathematical model predicted that sodium concentration decreases as the renal pressure increases, which is plausible because only water is reabsorbed at a rate inversely proportional to GFR at node N. At node K, the mathematical model predicted that sodium concentration decreases at all renal pressure levels with the same proportion, which is plausible because sodium reabsorption fraction is fixed regardless of the flow rate while water is not permeable at node K. At node E, where MD is located, the mathematical model predicted that faster flow is associated with less sodium reabsorption, and accordingly, higher sodium concentration, which is plausible because sodium reabsorption has an inversely proportional relationship with the flow rate at node E.

Figure 5B compares sodium concentration at different nodes at a normal renal arterial pressure (i.e., at $P_{RA}=$ 85 mmHg) predicted by the mathematical model to a simulation study by Layton and Layton (Layton and Layton, 2019). Our predictions were comparable to their predictions. In addition, our predictions were also comparable to Czerwin et al. (2021).

Figure 5C shows how UO drastically changes in response to renal arterial pressure variation, while GFR is relatively maintained by TGF and MM. Figure 5C also shows that the mathematical model can reproduce the phenomenon of pressure diuresis, e.g., for only a 50% increase in GFR, UO has a disproportionate increase of approximately 250%.

Figure 5D shows how renin release rate changes in response to renal arterial pressure variation. This nonlinear, inverse relationship is very similar to the behavior described by Kurtz (2012), and shows that renin secretion is inhibited in response to an increase in renal arterial pressure, which in turn lowers angiotensin production, and reverses the increase in renal arterial pressure and GFR.

### 3.2 Validation in pigs

Table 2 summarizes the accuracy metrics for CVP, CO, MAP, UO, and $\left[{Na}^{+}\right]$ predictions. Even though we simultaneously optimized the fit pertaining to five physiological variables (which often exposes the multi-objective optimization problem to trade-offs), all the predictions exhibited good tracking of their corresponding measured counterparts in terms of NMAE, correlation coefficient, and Bland-Altman limits of agreement (LoA). In addition, >91% of predicted and measured UO resided in the same operational range for pigs (<1 mL/h/kg, 1–1.5 mL/h/kg, and >1.5 mL/h/kg).

**TABLE 2:** Accuracy metrics for goodness of fit, calculated on an individual basis, pertaining to the mathematical model trained using the pig dataset. The metrics include normalized mean absolute error (NMAE; reported as median (IQR)), Pearson’s correlation coefficient (r), and Bland-Altman limits of agreement (LoA) reported as bias ± 2 × SD.

	CVP [mmHg]	CO [lpm]	MAP [mmHg]	UO [mL/h]	$\left[Na^{+}\right]$ [mEq/L]
NMAE [%]	14.2 (5.1)	16.4 (10)	14.43 (6.8)	15.11 (6.5)	15.61 (11)
r	0.86	0.79	0.84	0.74	0.81
LoA	−0.14 ± 3.05	0.04 ± 0.90	−0.91 ± 16.53	3.08 ± 32.34	0.21 ± 3.04

**FIGURE 6:**
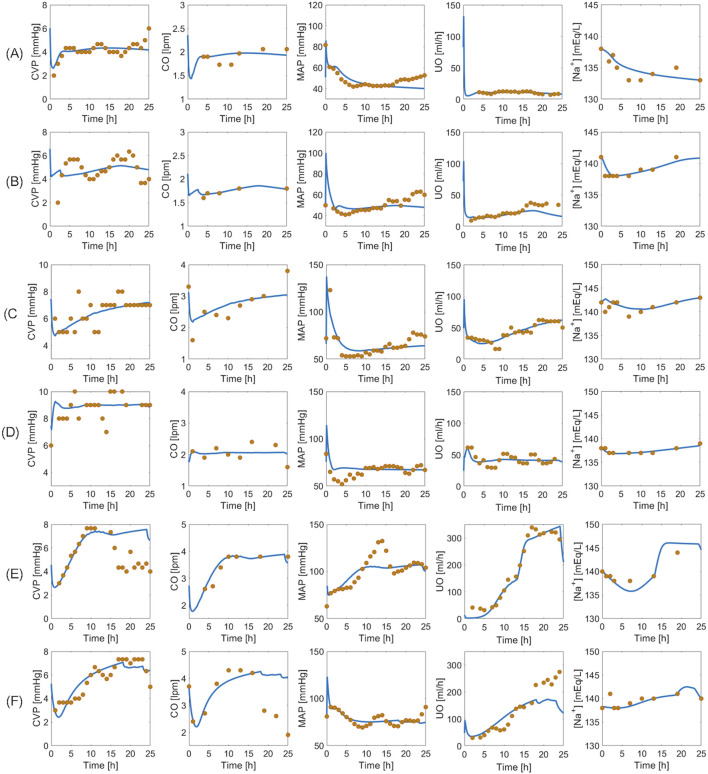
Validation in a total of six pig subjects. Each row represents an individual subject. **(A, B)**: Two subjects in un-resuscitated group (P1). **(C, D)**: Two subjects in adequately resuscitated group (P2). **(E, F)**: Two subjects in over-resuscitated group (P3). Solid blue line represents mathematical model prediction. Gold circles represent data.

Figure 6 provides visual examples of pig dataset and mathematical model predictions. Even within the same paradigm, there was substantial variability in how subjects responded to the injury and subsequent resuscitation. The mathematical model successfully replicated such variability. Figures 6A, B are two examples of the animals resuscitated with P1. While both animals showed signs of hypovolemia throughout the experiment as reflected by declines in CVP, CO, MAP, and UO, the intensity of the response to burn injury and the level of self-recovery offered by the body’s safety factors against hypovolemia and edema were different. For instance, the animal in Figure 6A experienced sharper ebbs in CVP, CO, MAP, and UO. The sodium plasma concentration trends also differed: continued decline in Figure 6A vs. recovery in Figure 6B. Figures 6C, D are two examples of the animals resuscitated with P2. While the animal in Figure 6C recovered to its baseline by the end of the experiment, the animal in Figure 6D showed signs of mild over-resuscitation. Figures 6E, F are two examples of the animals resuscitated with P3. Despite over-resuscitation, CVP in Figure 6E and CO in Figure 6F suggest modestly hypovolemic to normovolemic conditions toward the end of 24 h. The mathematical model could not replicate these behaviors, primarily because the animals were aggressively over-resuscitated with high doses of fluid, as reflected in their UO measurements. The mathematical model is not equipped with any mechanism to explain why CO and CVP were below normal levels in these animals. One possible explanation for the drop in CO in the subjects could be lowered heart rate, which the mathematical model is not equipped to predict. Another possible explanation could be measurement inaccuracy, which is plausible since CVP was measured with a central line, and CO via thermodilution using a Swan-Ganz catheter. However, all in all, the mathematical model successfully replicated most of the unique animal-specific responses while maintaining the expected trends associated with individual paradigms, as is evident from visual inspection of the plots as well as Table 2.

The pig dataset provided us with an unprecedented opportunity to train the mathematical model for large mammals resuscitated with vastly different protocols and test its capability to reflect the differences in burn resuscitation paradigms. To leverage this opportunity, we averaged the mathematical model simulations pertaining to each paradigm, and investigated the ability of the mathematical model to capture the between-group differences. In addition, to investigate the alignment of mathematical model simulations with established knowledge of physiology and burn pathophysiology, we likewise averaged the mathematical model simulations associated with the internal variables on kidney function and RAAS and examined their behaviors.

**FIGURE 7:**
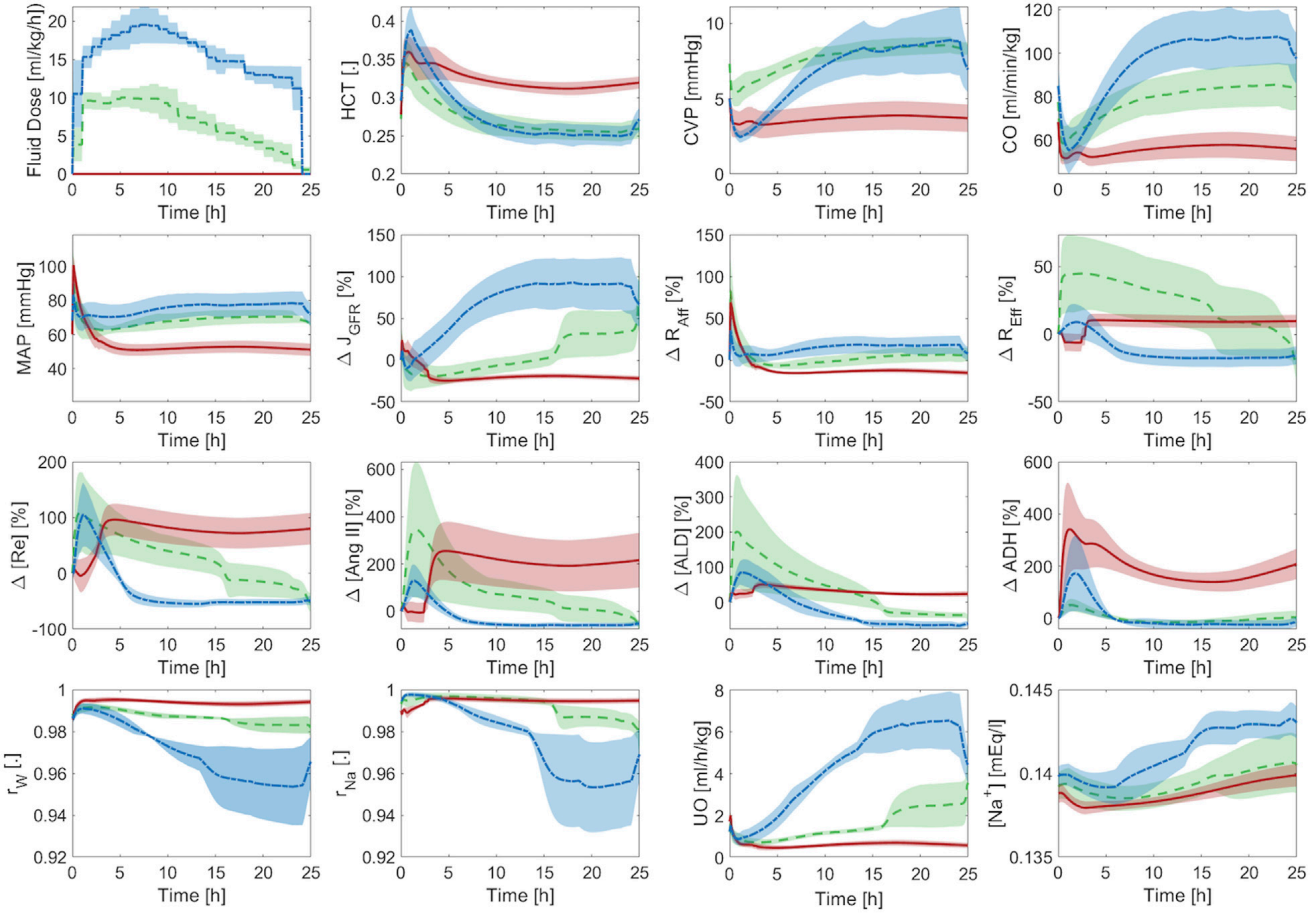
Validation in pigs: averaged mathematical model simulations. In each plot, red solid line shows the average prediction for the un-resuscitated group (P1), green dashed line shows the average prediction for the adequately-resuscitated group (P2), and blue dash-dotted line shows the average prediction for the over-resuscitated group (P3). The shaded areas represent standard errors. Fluid dose: scaled hourly infusion given to the animals. Δ : percentage deviation from the baseline value. $r_{W}$ : total water reabsorption fraction. $r_{\text{Na}}$ : total sodium reabsorption fraction.

Figure 7 shows the averaged mathematical model simulations to burn injury and resuscitation in pigs. Despite the large inter-subject variability reflected by the width of standard errors (shown as shaded areas) in some of the plots, the mathematical model was able to distinguish the responses pertaining to the three paradigms very well. Further, it could suggest possible physiological mechanisms responsible for their differences. The HCT increased immediately post-burn in all three paradigms, and while it stayed above baseline in P1, indicating hypovolemia, it decreased in both P2 and P3. In P2, the wide inter-subject variability in HCT (which encompassed both above and below the baseline level) suggests that while the average animal’s HCT was restored by the end of 24 h, some animals were still hypovolemic (HCT above baseline) or even ended up being over-resuscitated (HCT below baseline). The wide range of resuscitation outcome was also reflected by CO and CVP in P2. In P3, although the envelope was still wide, all the animals were considered over-resuscitated as the entire HCT envelop was below the baseline level, but with varying degrees of severity.

The mathematical model simulations of CO, CVP, ${\Delta J}_{GFR}$ , and UO for the three paradigms mirrored the behaviors expected from the corresponding HCT trends. Specifically, their initial decline due to hypovolemia (as HCT went up) continued to assume subnormal values in P1, a range of recovery from hypo-to hypervolemia in P2, and varying degrees of over-resuscitation in P3. The behavior of MAP, however, was different. While MAP remained below normal in P1, it could not distinguish P2 and P3 on its own, which agrees with studies suggesting that MAP is not a good stand-alone endpoint for fluid resuscitation (Oda et al., 2006).

Figure 7 also offers a comprehensive understanding of typical physiological responses to burn injury and resuscitation by virtue of the ability of our mathematical model to predict a wealth of internal physiological variables not typically available for routine measurement. When trauma and resuscitation alter $J_{GFR}$ , both afferent resistance ( $R_{Aff}$ ) and efferent resistance ( $R_{Eff}$ ) vary to regulate it. Despite the initial hypovolemia, $J_{GFR}$ initially increases due to a transient increase in MAP (vasoconstriction). Then, myogenic mechanism and TGF are triggered to counteract the increase in $J_{GF}$ by increasing the afferent resistance and decreasing the efferent resistance. As hypovolemia takes over in P1 indicated by a decrease in MAP and $J_{GFR}$ , renin, angiotensin, and aldosterone increase to (i) constrict the vessels and slow the decline in MAP, (ii) increase the efferent resistance and preserve $J_{GFR}$ , and (iii) increase the reabsorption fractions for sodium and water to retain more water and salt and correct the hypovolemic state of the body. ADH also increases in response to hypovolemia and contributes to the increase in water reabsorption fraction. In P2 and P3, these hormones and enzymes similarly increase initially to correct the hypovolemia induced by burn, but then decrease as the animals recover (P2) or get over-resuscitated (P3). $\left[{Na}^{+}\right]$ shows a wide range of behaviors in P1 and P2, but increases in P3 despite the decrease in aldosterone and total sodium reabsorption fractions. This is because the pure water reabsorption regulated by ADH decreases, which lowers total sodium concentration in plasma. This observation is consistent with Guyton’s finding that pure water reabsorption is a more effective determinant of $\left[{Na}^{+}\right]$ than aldosterone (Guyton, 1975), despite the fact that aldosterone is the direct actuator of sodium reabsorption fraction.

Supplementary Table S2 in Supplementary Material provides the mathematical model parameters for pigs. The estimated SS parameters were comparable to the values in the literature (when available), and therefore physiologically plausible.

### 3.3 Validation in sheep

**FIGURE 8:**
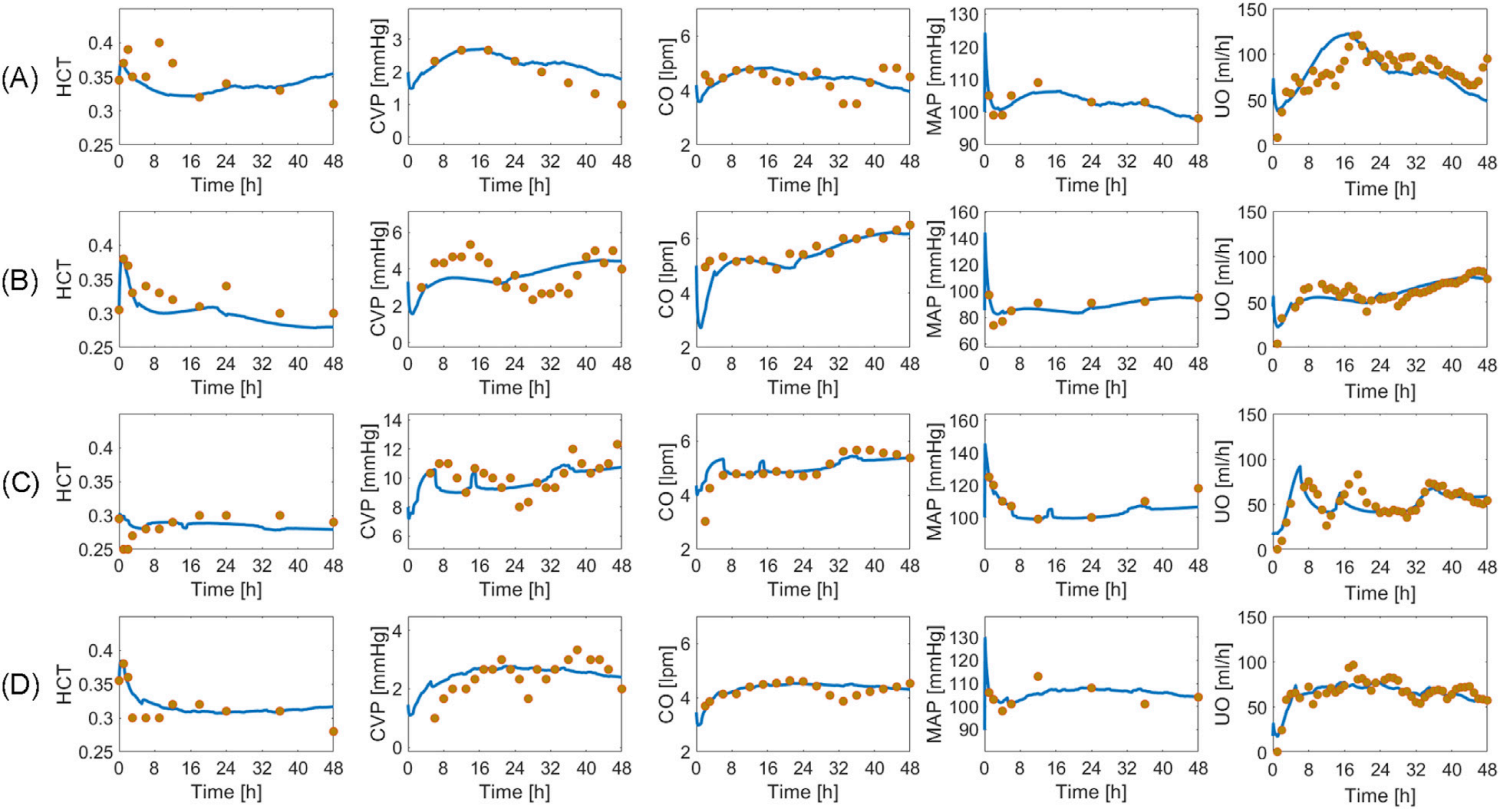
Validation in a total of four sheep subjects. Each row from **(A)** to **(D)** shows the predictions pertaining to a different sheep against its measured data. Solid blue line represents the prediction. Gold circles represent data.

Table 3 summarizes the goodness of fit metrics for HCT, CVP, CO, MAP, and UO predictions. The mathematical model performed consistently well, with small NMAE and strong correlations for all the variables. UO has the lowest correlation with the experimental data. Admittedly, 0.55 is only a moderate correlation. Yet, 76% of predicted and measured UO resided in the same operational range for sheep (<0.5 mL/h/kg, 0.5–1.0 mL/h/kg, and >1.0 mL/h/kg) on the average. This is an encouraging performance given that current burn resuscitation protocols adjust resuscitation dose based on UO range rather than its absolute value. Further, the mathematical model predicted all the variables while being characterized with physiologically acceptable parameter values (Supplementary Table S2 in Supplementary Material). Figure 8 visually depicts how the mathematical model can predict HCT, CVP, CO, MAP, and UO with reasonable accuracy and capture the inter-subject variability among animals, even those subject to similar resuscitation protocols and injury severity.

**TABLE 3:** Accuracy metrics for goodness of fit, calculated on an individual basis, pertaining to the mathematical model trained using the sheep dataset. The metrics include normalized mean absolute error (NMAE; reported as median (IQR)), Pearson’s correlation coefficient (r), and Bland-Altman limits of agreement (LoA) reported as bias±2 × SD.

	HCT	CVP [mmHg]	CO [lpm]	MAP [mmHg]	UO [mL/h]
NMAE [%]	16.8 (13.4)	19.1 (8.7)	13.4 (8.6)	16.1 (8.4)	15.7 (6.1)
r	0.85	0.62	0.83	0.83	0.55
LoA	0.003 ± 0.05	−0.18 ± 2.5	0.1 ± 0.97	−1.96 ± 12.75	−3.8 ± 45

**FIGURE 9:**
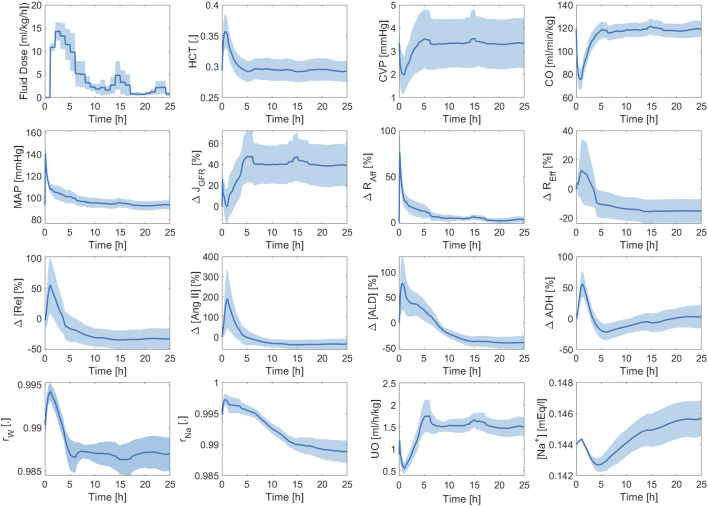
Validation in sheep: averaged mathematical model simulations. Solid line shows average prediction. Shaded area shows standard error. Fluid dose: scaled hourly infusion given to the animals. Δ : percentage deviation from the baseline value. $r_{W}$ : total water reabsorption fraction. $r_{Na}$ : total sodium reabsorption fraction.

Figure 9 shows the averaged mathematical model simulations to burn injury and resuscitation associated with CV, RAAS, and kidney function variables. HCT increases immediately post-burn due to hypovolemia, which is also reflected by a drop in CVP and CO. All three variables recover to their respective baseline values, and even show signs of over-resuscitation, which is consistent with the experimental data (see Figure 8). In contrast, MAP increases immediately post-burn despite the decrease in CO, which may be due to the vasoactive effects of inflammatory agents. However, MAP eventually decreases back to its baseline level on the average. The transient increase in MAP causes a transient increase in GFR and UO. But, they almost immediately decrease down to sub-normal values due to hypovolemia. Finally, they increase to supra-normal values as the animals undergo mild over-resuscitation. GFR shows a sustained increase compared to the other variables, because it is also affected by plasma albumin dilution (Zdolsek et al., 2010). As a result, the efferent resistance decreases while the afferent resistance increases, which collectively restore GFR to its normal level. As expected, all three elements of RAAS increase in response to the initial decrease in GFR to aggressively retain water and sodium. But, they eventually subside to remove excess water from the body and to lower BP, which is reflected in the decrease in total sodium reabsorption fraction. The decrease in total water reabsorption fraction is a result of the decrease in both RAAS activity and ADH level as the animals become slightly over-resuscitated. As a result, UO increases and remains above its normal range.

### 3.4 Validation in humans

Based on the sensitivity analysis described in Section 2.3.3, we determined 10 SS parameters (see Supplementary Table S2, Supplementary Material). Then, we validated the mathematical model using the training and test datasets.

**FIGURE 10:**
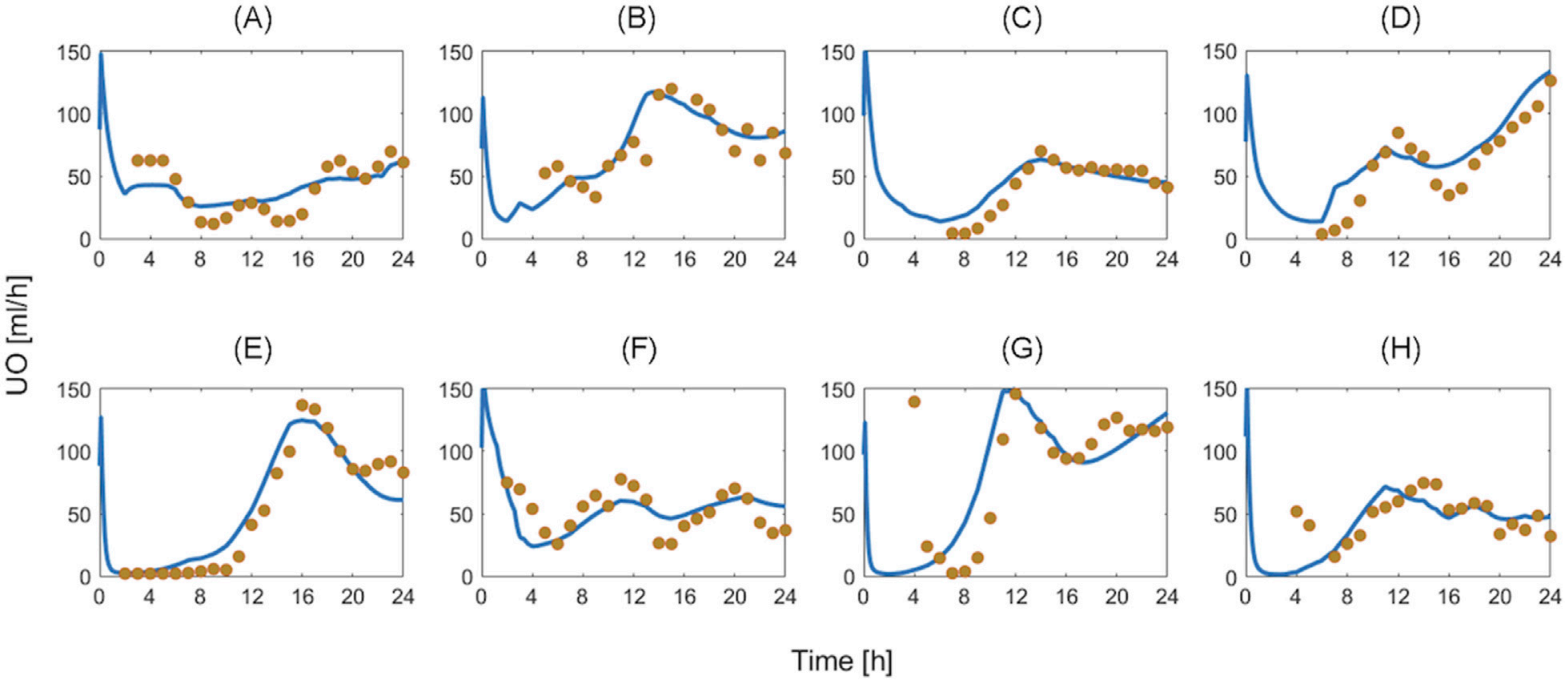
Validation in humans: eight exemplary burn patients with diverse weights and burn injury severities. Each plot shows measured urinary output (UO) responses associated with a burn patient vs. the UO responses predicted by the mathematical model. Solid blue line denotes prediction. Gold circles denote data. The patient demographics are as follows: **(A)** TBSA 27% with 80 kg weight. **(B)** TBSA 36% with 66 kg weight. **(C)** TBSA 46% with 90 kg weight. **(D)** TBSA 60% with 71 kg weight. **(E)** TBSA 24% with 81 kg weight. **(F)** TBSA 35% with 94 kg weight. **(G)** TBSA 50% with 89 kg weight. **(H)** TBSA 60% with 102 kg weight.

Table 4 summarizes the goodness of fit metrics for UO predictions, which show that the mathematical model predicted UO in real burn patients very well in both training and test datasets. For example, NMAE in the test dataset is almost one-third of the prediction errors pertaining to a recent work based on a black box model to predict UO (NMAE = 30 (Meng et al., 2015)%) (Luo et al., 2015). This is a very encouraging improvement, especially considering the fact that our mechanistic mathematical model could predict many other internal physiological variables consistently with the literature, and with plausible parameter values (see Supplementary Table S2, Supplementary Material). Figure 10 shows that the mathematical model, even after restricting SS parameters to 10, could reproduce the inter-individual variability among patients with very similar demographics and injury severities. Each column in Figure 10 shows two patients whose weight and TBSA are almost identical. Despite the similarities, the response to burn injury and resuscitation were notably different. Yet, the mathematical model could replicate the physiological differences.

**TABLE 4:** Accuracy metrics for goodness of fit, calculated on an individual basis, pertaining to the mathematical model trained using the human dataset. The metrics include normalized mean absolute error (NMAE; reported as median (IQR)), Pearson’s correlation coefficient (r), and Bland-Altman limits of agreement (LoA) reported as bias ± 2 × SD.

	Training (N = 120)	Test (N = 113)
NMAE [%]	13.15 (5.5)	11.20 (6.0)
r	0.69	0.85
LoA	0.94 ± 43	0.34 ± 36

**FIGURE 11:**
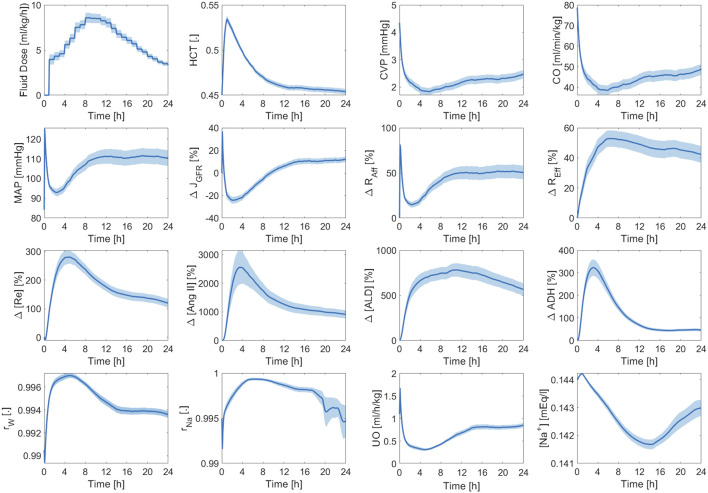
Validation in humans: averaged mathematical model simulations (120 patients in the training dataset). Solid line shows average prediction. Shaded area shows standard error. Fluid dose: scaled hourly infusion given to the patients. Δ : percentage deviation from the baseline value. $r_{W}$ : total water reabsorption fraction. $r_{Na}$ : total sodium reabsorption fraction.

Figure 11 shows the averaged mathematical model simulations to burn injury and resuscitation pertaining to the training dataset. On the average, the patients in the training dataset remained hypovolemic at the end of the 24 h, which is reflected by supra-baseline HCT values and sub-baseline value CVP and CO values. MAP was predicted to be higher than baseline for the entire 24 h due to vasoconstriction, which again demonstrates that it is not a good indicator of fluid resuscitation on its own, in accordance with the literature (Oda et al., 2006). Despite the decrease in CO, GFR remained supra-normal due to the dilution of plasma albumin as confirmed by experiments (Zdolsek et al., 2010). All three components of the RAAS were still supra-normal at the end of 24 h. If we continue the simulation beyond this point, we will observe a sustained decline in RAAS elements until a normal MAP is restored. The total reabsorption fractions for water and sodium increased and remained at supra-baseline levels to retain more water and replace the lost PV. However, the plasma sodium concentration was slightly sub-normal due to the increase in water reabsorption.

## 4 Conclusion

Previously, we developed and extensively validated mathematical models of burn injury and resuscitation using experimental data obtained from sheep and human burn patients. Here, we improved and extended the original mathematical model, particularly for kidney function and electrolyte dynamics by adding a detailed, mechanistic mathematical model of the kidney’s intrinsic regulatory mechanisms as well as reabsorption mechanisms. Additionally, motivated by the recent interests in hemodynamic monitoring of burn patients, we integrated CV system into the mathematical model. We also added RAAS which is directly relevant to both kidney function and CV system.

Using the experimental data collected from 15 pigs resuscitated with 3 distinct protocols, 9 sheep, and 233 humans, all with extensive burns, we validated the new components of the mathematical model and showed that it could not only predict HCT, CVP, CO, MAP, UO, and $\left[Na^{+}\right]$ with an adequate accuracy, but also provide insights into the burn resuscitation effectiveness in restoring VK, CV, and kidney function variables.

To the best of our knowledge, this is the first study to show the potential to conduct hypothesis testing relevant to burn injury and resuscitation using a mathematical model, which is extensively validated for VK, CV, kidney function, and RAAS mechanisms in a large and diverse set of burn patients with significant inter-subject variability.

The validation of our mathematical model has several limitations. First, validation of our human mathematical model was limited to urinary output measurements. To address this shortfall, we extensively investigated the plausibility of the predicted variables against available literature. However, literature cannot entirely substitute more comprehensive datasets. Second, since all burn patients and experimental subjects in our dataset were resuscitated with LR, the mathematical model may lack validated predictions for other resuscitation fluids, such as colloids or alternative crystalloids, limiting its applicability in diverse clinical scenarios.

In the future, we may use the mathematical model to generate cohorts of virtual patients suited to *in silico* testing of new burn resuscitation protocols and decision support systems. In pre-clinical settings based on large animals, we may use the mathematical models trained and tested using pigs and sheep. These large mammals are regularly used for pre-clinical *in vivo* testing due to several advantages over rodents. However, all these experiments are resource intensive, time-consuming and impose financial and ethical costs (Burmeister et al., 2022). A credible and transparent mathematical model could essentially complement large animal experiments in the pre-clinical development and evaluation of new resuscitation algorithms by way of its ability to furnish plausible and realistic predictions of physiological variables in response to burn injury and resuscitation.

In addition to the intended use of the mathematical model as a reliable *in silico* evaluation platform, the mathematical model may offer additional benefits, including: (i) serving as a training tool for healthcare providers, (ii) a scientific tool for garnering deep insight into the pathophysiology of burn shock and resuscitation which cannot be gathered from animal experiments and clinical studies, and (iii) a mathematical tool to enable the simulation of CV system, kidney function, and the RAAS targeted to purposes and contexts beyond burn injury and resuscitation.

## Data Availability

The data analyzed in this study is subject to the following licenses/restrictions: The sources of data for the pigs, sheep, and human subjects are referenced in the paper. None of the datasets were publicly accessible, but co-authors involved in collecting the data had access and permission to use it for this analysis. Requests to access these datasets should be directed to david.burmeister@usuhs.edu for pig data, george.kramer@gmail.com for sheep data, andjose.salinas4.civ@mail.mil for the clinical dataset.
