# Supplementary material for: A mathematical model for simulation of cardiovascular, renal, and hormonal responses to burn injury and resuscitation
Source: Front Physiol. 2024 Oct 3;15:1467351. doi: 10.3389/fphys.2024.1467351 (PMC11484069; doi:10.3389/fphys.2024.1467351)
Supplement: Supplementary file 1 [file DataSheet1.docx]

**Supplementary Material**

Section 2.1 and Fig. 1 in the paper describe the general model structure and schematic. Sections S1-S4 provide all the mathematical details of the mathematical model components described in section 2.1 of the manuscript. Abbreviations and mathematical model parameters follow in Table S1 and Table S2, respectively.

**S1. Volume Kinetics and Burn-Induced Perturbations**

The mathematical model of volume kinetics (VK) and burn-induced perturbations were developed and validated using sheep burn subjects and human clinical data in our prior work [1], [2]. Section 2.1.1 provides an overview of the VK and burn-perturbation assumptions. Volume kinetics was modeled using a classical multi-compartmental approach, with three separate compartments representing the vasculature, intact tissues, and burned tissues, containing water and albumin as a representative of all blood protein. The water and albumin balances within each compartment were derived based on the principle of mass conservation, and primarily regulated by a bi-directional filtration across the capillary membrane and by lymphatic vessels which collect and return the excessive fluid to plasma. Subjects are assumed to lose fluid from plasma by urinary output (UO), and by evaporation and exudation (through burn wounds) from skin. They can gain fluid via intravenous fluid infusion, and through protein denaturation, i.e., an influx of albumin into the burnt tissues after collagen denaturation due to heat. Based on above we used the following ordinary differential equations to describe the water and albumin balance in model compartments [3]:

| $\frac{{d(V}_{P})}{dt}={-J}_{C,BT}-J_{C,IT}+J_{L,BT}+J_{L,IT}+J_{I}-J_{UO}$, | (S1) |
| --- | --- |
| $\frac{d\left( V_{BT} \right)}{dt}=J_{C,BT}-J_{L,BT}-J_{EX}-J_{EV,BT}$, | (S2) |
| $\frac{d\left( V_{IT} \right)}{dt}=J_{C,IT}-J_{L,IT}-J_{EV,IT}$, | (S3) |
| $\frac{d\left( A_{P} \right)}{dt}={-Q}_{C,BT}-Q_{C,IT}+Q_{L,BT}+Q_{L,IT}+Q_{I}$, | (S4) |
| $\frac{d\left( A_{BT} \right)}{dt}=Q_{C,BT}-Q_{L,BT}-Q_{EX}+Q_{PD}$, | (S5) |
| $\frac{d\left( A_{IT} \right)}{dt}=Q_{C,IT}-Q_{L,IT}$, | (S6) |

in this formulation $V$ represents water volume, $A$ stands for albumin content, $J$ is water flow, and $Q$ denotes albumin flow. The subscripts $P$, $\mathrm{BT}$, $\mathrm{IT}$ stand for plasma, burnt tissues, and intact tissues. The subscripts $C$, $L$, $I$, $U$, $EV$, $EX$, and $PD$ correspond to capillary filtration, lymphatic flow, fluid infusion, UO, evaporation, exudation, and protein denaturation.

The Starling equations which characterize the net movement of water across capillary membrane by balancing the hydrostatic and colloid oncotic pressures were used to determine the capillary filtration rates:

| $J_{C,X}=K_{C,X}\left[ P_{C}-P_{X}-\sigma_{X}\left( \pi_{C}-\pi_{X} \right) \right]$, | (S7) |
| --- | --- |
| $\pi_{C}=C_{O}\left[ A \right]_{P}, \pi_{X}=C_{O}\left[ A \right]_{X}$*,* $\left[ A \right]_{P}=\frac{A_{P}}{V_{P}}, \left[ A \right]_{X}=\frac{A_{X}}{V_{X}}$, | (S8) |

where $X\in\left\{ BT, IT \right\}$, $P_{C}$ and $P_{X}$ represent capillary and tissue hydrostatic pressures, while $\pi_{C}$ and $\pi_{X}$ are plasma and tissue colloid oncotic pressures. $K_{C,X}$ and $\sigma_{X}$ represent capillary filtration and albumin reflection coefficients, respectively. $\left[ A \right]_{P}$ and $\left[ A \right]_{X}$ are the albumin concentrations in plasma and tissue, respectively, with $C_{O}$ being the colloid oncotic pressure constant. The excessive water filtered from plasma is returned by the lymphatic flow, modeled using a phenomenological approach and represented as a sigmoidal curve [2]:

| $J_{L,X}= \frac{\bar{J}_{L,X}}{C_{L}+\left( 1-C_{L} \right)e^{-S_{L}\left( P_{X}-\bar{P}_{X} \right)}}$, | (S9) |
| --- | --- |

Here $\bar{J}_{L,X}$ is nominal lymphatic flow at nominal tissue hydrostatic pressure, $\bar{P}_{X}$. Constants $C_{L}$ and $S_{L}$ respectively denote the inverse of the maximum possible increase in lymphatic flow, and the lymphatic sensitivity to changes in tissue hydrostatic pressure. The lymphatic albumin flow is influenced by both the lymphatic water flow and the albumin concentration in the corresponding tissue:

| $Q_{L,X}=J_{L,X}\left[ A \right]_{X}$, | (S10) |
| --- | --- |

A coupled diffusion-convection equation by Bresler et al [4] was used to estimate albumin transport through capillary pores:

| $Q_{C,X}=J_{C,X}\left( 1-\sigma_{X} \right)\left\{ \frac{\left[ A \right]_{P}-\left[ A \right]_{X}e^{-\frac{\left( 1-\sigma_{X} \right)J_{C,X}}{PS_{X}}}}{1-e^{-\frac{\left( 1-\sigma_{X} \right)J_{C,X}}{PS_{X}}}} \right\}$, | (S11) |
| --- | --- |

where $\mathrm{PS}$ is permeability-surface area coefficient. To fully characterize capillary filtration and lymphatic flow, $P_{C}$ was described as a linear function of $V_{P}$:

| $P_{C}=\bar{P}_{C}+E_{C}\left( V_{P}-\bar{V}_{P} \right)$, | (S12) |
| --- | --- |

Here $\bar{P}_{C}$ is the nominal capillary hydrostatic pressure associated with the nominal PV ($\bar{V}_{P}$), and $E_{C}$ represents capillary elastance. For the interstitial compartments, we applied the nonlinear pressure-volume model developed by Øien and Wiig [5] at the microscopic level and adapted it for use in a macroscopic context:

| $P_{X}=-\frac{\alpha}{R}+\gamma$, | (S13) |
| --- | --- |
| $R=R\left( y_{X} \right)=\hat{R}\left[ 1-\left( 1-\beta\right)(\frac{y_{X}-\hat{y}}{\check{y}-\hat{y}}) \right]^{n}$, | (S14) |
| $y_{X}=0.75\left( 1+\bar{W}_{X}\frac{V_{X}}{\bar{V}_{X}} \right)$, | (S15) |

where $\alpha$, $\gamma$,$\hat{R}$ , $\beta$, and $n$ are constant coefficients, $y_{X}$ represents the hydration in tissue $X$ at the microscopic scale, with $\hat{y}$ and $\check{y}$ denoting its maximum and minimum values, respectively. *R* is the radius of glycosaminoglycans, which varies in response to $y_{X}$ variation. Eq. (S15) relates microscopic hydration to macroscopic hydration, where $\bar{W}_{X}$ is the nominal hydration level, defined as the ratio of the water volume in $X$ to its dry weight.

The new additional development to VK in this work is sodium dynamics, which is enabled by the integration of a detailed mathematical model of renal function and hormonal system. Since both the capillary filtration and lymphatic flows are isotonic, we assume that sodium content in the extracellular fluid (ECF) is altered only by sodium gain through sodium fluid infusion rate ($F_{I}$) and sodium excretion rate through urinary output (UO; $F_{UO}$):

| $\frac{d\left( Na_{ECF}^{+} \right)}{dt}=F_{I}-F_{UO}$, | (S16) |
| --- | --- |
| $\left[ Na^{+} \right]=\left[ Na^{+} \right]_{ECF}=\frac{Na_{ECF}^{+}}{V_{P}+V_{BT}+V_{IT}}$, | (S17) |

Where F represents sodium flow, $Na_{ECF}^{+}$ is the total sodium content in the extracellular fluid (ECF) and $\left[ Na^{+} \right]_{ECF}$ is sodium concentration in the ECF, which is the same as the plasma sodium concentration. Any change in $\left[ Na^{+} \right]_{ECF}$ due to exchanges with the intracellular fluid are assumed to happen faster than our simulation time step of interest. $F_{I}$ is taken from datasets as input, and $F_{UO}$ is determined in Section S3.2.

A universal function was designed to model the various transient and highly individualized perturbations induced by burn injury:

| $\phi\left( M_{W},\lambda_{1,W},\lambda_{2,W},t \right)=M_{W}\left( e^{-\lambda_{1,W}t}-e^{-\lambda_{2,W}t} \right)$, | (S18) |
| --- | --- |

where $M_{W}$ represents the perturbation value at peak, and $\lambda_{1,W}$ and $\lambda_{2,W}$ are the slow and fast time constants, all corresponding to a perturbation $W\in\left\{ \alpha_{BT},\alpha_{IT},P_{C},P_{BT}, MAP \right\}$, where $\alpha_{BT}$ and $\alpha_{IT}$ are the pore radius ratios associated with burnt and intact tissues (see below for details). Eq. (S18) describes a post-burn perturbation which reaches $M_{W}$ at a varying time after the injury and then begins to decay at a rate specified for perturbation W, often found by fitting model predictions to data (Section 2.2.3). Details for perturbations related to VK follow.

The destruction of capillaries in burnt tissues is expressed as perturbations in the capillary filtration coefficient $K_{C,BT}$ and permeability surface area coefficient $PS_{BT}$. We expressed capillary filtration, albumin reflection, and permeability-surface area coefficients associated with burnt and intact tissues as functions of the pore ratios $\alpha_{X}$, $X\in\left\{ BT, IT \right\}$, defined as the ratio of the albumin radius to the capillary pore radius [1], [2], [6], [7]:

| $K_{C,X}=\bar{K}_{C,X}k_{PD,X}\frac{\bar{\alpha}_{X}^{4}}{\alpha_{X}^{4}}$, | (S19) |
| --- | --- |
| $\sigma_{X}=1-\left( 1-\alpha_{X} \right)^{2}$, | (S20) |
| $S_{X}=\bar{PS}_{X}k_{PD,X}\frac{\bar{\alpha}_{X}^{2}\left( 1-\alpha_{X}^{2} \right)}{\left( 1-\bar{\alpha}_{X}^{2} \right)\alpha_{X}^{2}}$, | (S21) |

where $k_{PD,X}$ is the remaining fraction of the capillaries after partial destruction, and $\bar{K}_{C,X}$, $\bar{PS}_{X}$, and $\bar{\alpha}_{X}$ are nominal values of $K_{C,X}$, $PS_{X}$, and $\alpha_{X}$ adjusted for the water fraction in burnt and intact tissues, weight, and capillary recruitment [3]:

| $\bar{K}_{C,BT}=\bar{K}_{C}\varepsilon_{B}r_{FV}\eta_{CR}, \bar{K}_{C,IT}=\bar{K}_{C}\left( 1-\varepsilon_{B}r_{FV} \right)\eta_{CR}$, | (S22) |
| --- | --- |
| $\bar{PS}_{BT}=\bar{PS}\varepsilon_{B}r_{FV}\eta_{CR}, \bar{PS}_{IT}=\bar{PS}\left( 1-\varepsilon_{B}r_{FV} \right)\eta_{CR}$, | (S23) |

Here $\bar{K}_{C}$ and $\bar{PS}$ are nominal capillary filtration and permeability surface area coefficients before burn injury, $\eta_{CR}=\left( 2\frac{V_{P}}{\bar{V}_{P}}-1 \right)$, and $r_{FV}$ is the ratio of fluid volume in skin to total interstitial compartment [3]. After burn injury happens, Eq (S18) is applied to the capillary pore radius ratios and consequently “perturbs” capillary coefficients as dictated by Eq. (S19-S21):

| $\alpha_{X}\left( t \right)=\bar{\alpha}_{X}-\phi(M_{\alpha_{X}},\lambda_{1,\alpha_{X}},\lambda_{2,\alpha_{X}},t)$, | (S24) |
| --- | --- |
|  |  |

The transient negative hydrostatic pressure in burn tissues followed the same form:

| $\Delta P_{BT}\left( t \right)=-\phi\left( {M_{P}}_{BT}, \lambda_{1,P_{BT}},\lambda_{2,P_{BT}},t \right)$, | (S25) |
| --- | --- |

Which is then added to Eq (S13). The increase in capillary hydrostatic pressure due to vasodilation was expressed the same, and added to Eq. (S12):

| $\Delta P_{C}\left( t \right)=\phi(M_{P_{C}},\lambda_{1,P_{C}},\lambda_{2,P_{C}},t)$, | (S26) |
| --- | --- |

The albumin influx due to the heat-generated protein denaturation Eq (S5) in burnt tissues was assumed to peak immediately post-burn, and undergo an exponential decay:

| $Q_{PD}=\hat{Q}_{PD}e^{-\lambda_{PD}t}$, | (S27) |
| --- | --- |

Our final perturbation, a new addition to perturbation model in this work, is a transient vasoconstriction which leads to an increase in MAP despite lowered PV, described in Section S2, Eq. (S41).

Finally, evaporation and exudation rates were estimated from literature, adjusted by weight and total burned surface areas (TBSA) [8], [9]:

| $J_{EV,BT}=\left\{ \begin{aligned} K_{1,EV}{\varepsilon_{B}S_{B}e}^{\lambda_{1,EV}t}, t<6 hr \\ K_{2,EV}{\varepsilon_{B}S_{B}e}^{\lambda_{2,EV}t}, t>6 hr \end{aligned} \right.$, | (S28) |
| --- | --- |
| $J_{EV,IT}=K_{1,EV}{(1-\varepsilon}_{B})S_{B}$, | (S29) |
| $S_{B}=0.0242H^{0.396}W^{0.538}$, | (S30) |
| $J_{EX}=K_{EX}\varepsilon_{B}S_{B}e^{\lambda_{EX}t}$, | (S31) |
| $Q_{EX}=J_{EX}\eta_{EX}\left[ A \right]_{BT}$, | (S32) |

where $K_{1,EV},K_{2,EV}$, $K_{EX}$, $\lambda_{1,EV}$, $\lambda_{2,EV}$, and $\lambda_{EX}$ are constant coefficients, $\varepsilon_{B}$ is the fraction of body surface area subject to burn, $S_{B}$ is total body surface area based on the Haycock formula, and $\eta_{EX}$ is the ratio between albumin concentration in the exudate and albumin concentration in the burnt tissues [3].

**S2. Cardiovascular Physiology**

Section 2.1.2 presents an overview of the model. We modeled the relationship between BV and MSP as follows (Fig. 2(A)):

| $MSP=MSP_{0}\times e^{\lambda_{MSP}\left( V_{B}-V_{B0} \right)}$ | (S33) |
| --- | --- |

where $MSP_{0}$ is nominal value of MSP (see Eq. (S42)), $\lambda_{MSP}$ represents the sensitivity of MSP to BV, and $V_{B0}$ is nominal value of BV. Note that BV is calculated in the mathematical model of VK. This mathematical model relating MSP to BV is based on recent studies indicating that the relationship between BV and MSP may be exponential rather than linear (as conventionally assumed [10], [11]). Strictly, MSP depends on venous BV. However, the use of entire BV is a simplification that is acceptable except for very low values of CO.

TPR is a primary regulator of acute BP in the body and is mainly modulated by ANS and the hormonal system, particularly angiotensin II (Ang II). Since ANS-modulated changes in TPR are short-term (in the order of minutes) and may be insignificant in the time scale relevant to burn injury and resuscitation (>24 hours), we primarily focus on the effects of Ang II [12], [13]. According to the experimental data obtained from humans, the relationship between TPR and the plasma concentration of Ang II may be best approximated by a sigmoid function [14] (Fig. 2(B)):

| $TPR=\frac{{(1+K}_{TPR})\times TPR_{0}}{1+K_{TPR}\times e^{-\lambda_{TPR}(\left[ Ang II \right]-\left[ Ang II \right]_{0})}}$, | (S34) |
| --- | --- |

where $TPR_{0}$ is nominal value of TPR, $\left[ Ang II \right]$ is the plasma concentration of Ang II with its nominal value $\left[ Ang II \right]_{0}$, and $K_{TPR}$and$\lambda_{TPR}$ are constant coefficients (see Section **S4.1** for more details).

VR is proportional to the pressure drop across systemic veins, and is inversely related to return venous resistance (RVR) [15]:

| $VR=\frac{MSP-RAP}{RVR}=\frac{MSP-CVP}{RVR}$*,* | (S35) |
| --- | --- |

where RAP is right atrial pressure, which is assumed to be equal to CVP. RVR is assumed to be equal to 7% of the TPR at any instant [16]:

| $RVR=0.07\times TPR$*.* | (S36) |
| --- | --- |

On the other hand, CO is proportional to CVP in what could be described as a series of dose-response curves whose maxima depend on cardiac effectiveness (CE):

| $CO=\frac{CO_{max}(CE)}{1+\frac{{CVP}_{50}}{CVP}}$, | (S37) |
| --- | --- |

where $CO_{max}(CE)$ is maximum CO, and $CVP_{50}$is CVP at which CO will reach 50% of its maximum value.

In the steady-state, CO and VR must be equal to each other according to the Frank-Starling law, and their true value could be found by finding the intersection point (i.e., circulatory equilibrium) on the CO-VR curve (Fig. 2(C)). However, this is complicated by the effect of the CE on the CO curve. Hence, in many cases, an iterative approach is required to find the operating CVP point. To simplify the mathematical model of CO-VR curve and facilitate finding an analytical solution for CVP, we ignore the effect of CE on CO. This simplifying assumption is supported by Kenji [17] and Guyton et al. [18], [19], who illustrated that for a normal heart equilibrium CO estimated by the CE-adjusted CO curve is very close to that of an unadjusted CO curve. Therefore, we can assume $CO_{max}$ to be a constant coefficient:

| $CO_{max}=CO_{0}\times M_{CO}$, | (S38) |
| --- | --- |
|  |  |

where $M_{CO}$ is a constant coefficient representing maximal increase in CO. Now, we can find the operating CVP by equating Eq. (S35) to (S37):

| $CVP=\frac{1}{2}\left[ MSP-CVP.CO_{max}-CVP_{50}\pm\left( MSP^{2}+RVR^{2}\cdot CO_{max}^{2}+CVP_{50}^{2}-2\times MSP(RVR\cdot CO_{max}-CVP_{50})+2RVR\cdot CO_{max}CVP_{50} \right)^{\frac{1}{2}} \right].$ | (S39) |
| --- | --- |

This yields two solutions for CVP, among which only the larger, which is non-negative, is plausible. Having CVP, CO can be calculated from either Eq. (S35) or Eq. (S37).

Using CO and TPR, we modeled MAP as follows:

| $MAP=CO\times TPR-CO_{0}\times TPR_{0}+MAP_{0}$*,* | (S40) |
| --- | --- |

where $CO_{0}$, $TPR_{0}$, and $MAP_{0}$ are nominal CO, TPR, and MAP, respectively.

Burn injury elicits a surge of inflammatory responses which results in co-existing vasoconstriction and vasodilation [20], [21]. Vasoconstriction, which happens in large vessels, results in a transient increase in MAP despite lowered BV and CO [22]. Indeed, the transient increase in MAP was consistently observed in our pig and sheep datasets. To capture this transient behavior, we introduced a term representing burn-induced perturbation in MAP to Eq. (S40) (Fig. 2(D)):

| $MAP=CO\times TPR-CO_{0}\times TPR_{0}+MAP_{0}+ M_{MAP}\left( e^{-\lambda_{1,MAP,}t}-e^{-\lambda_{2,MAP}t} \right)$*,* | (S41) |
| --- | --- |

where $M_{MAP}$ is maximum transient increase in MAP, and $\lambda_{1,MAP}$ and $\lambda_{2,MAP}$ are slow and fast time constants associated with the decay of the perturbation in MAP.

We considered two constraints to ensure that nominal CO and CVP values are physiologically plausible in all the datasets we used in this work. To ensure that TPR is always positive, we assumed that nominal MSP is 5mmHg larger than nominal CVP:

| $MSP_{0}=CVP_{0}+5.$ | (S42) |
| --- | --- |

In addition, to ensure that nominal CO is obtained at nominal CVP, we used the following value of ${CVP}_{50}$:

| ${CVP}_{50}=CVP_{0}(\frac{CO_{max}}{CO_{0}}-1)$. | (S43) |
| --- | --- |

**S3. Renal Function**

**S3.1. Glomerular Filtration Rate and Renal Plasma Flow Regulation**

Following Poiseuille’s Law, the rate at which plasma flows into the kidneys must be directly proportional to the pressure drop across the kidneys and inversely proportional to total renal resistance:

| $J_{RPF}=\frac{P_{RA}-P_{RV}}{R_{Kidney}}$*,* | (S44) |
| --- | --- |
| $R_{Kidney}=R_{Aff}+R_{Eff}+R_{RV}$, | (S45) |

where $P_{RA}$ is the renal arterial pressure, $P_{RV}$ the renal venous pressure, and $R_{Kidney}$is the total renal resistance, which consists of the renal afferent resistance ($R_{Aff}$), the renal efferent resistance ($R_{Eff}$), and the renal venous resistance ($R_{RV}$). The baseline value of total renal resistance is chosen such that a 70 kg person would have a normal 625 ml/min RPF [23], and this resistance is distributed across the kidneys to yield plausible initial values for different components of the renal resistance according to literature (Table S2).

$P_{RA}$ and $P_{RV}$ are linear functions of MAP and CVP, respectively, with plausible initial values [16], [24]:

| $P_{RA}=85+K_{RA}(MAP-MAP_{0})$, | (S46) |
| --- | --- |

| $P_{RV}=3+K_{RV}(CVP-CVP_{0})$, | (S47) |
| --- | --- |

where $K_{RA}$ and $K_{RV}$ are constant coefficients.

The myogenic mechanism (MM) and the Tubuglomerular feedback mechanism (TGF) are the two most important regulatory mechanisms in the kidneys, whose primary actuator is the afferent resistance. Therefore, the afferent resistance is assumed to be comprised of three parts:

| $R_{Aff}=R_{MM}+R_{TGF}+R_{A,0}$, | (S48) |
| --- | --- |

where $R_{MM}$ is the part regulated by MM, $R_{TGF}$ is the part of the afferent resistance regulated by TGF, and $R_{A,0}$ is a constant, baseline resistance.

MM is sensitive to changes in the renal arterial pressure, and modulates the afferent resistance to maintain RPF and GFR. To estimate $R_{MM}$, we used a scaled version of the following equation derived by Moore et al. [25], and tested by Moss et al. [26], and Czerwin et al. [24]:

| $R_{MM}=G_{MM}\left( R_{TGF}+R_{A,0}+R_{Eff} \right)H(\frac{P_{RA}}{P_{ref}}-1)$*,* | (S49) |
| --- | --- |

where $G_{MM}$ is the MM scaling coefficient chosen to yield plausible initial values for $R_{MM}$ (Table S2). The Heaviside function ($H$) ensures that $R_{MM}$ is zero below a reference pressure $P_{ref}$, and that $R_{MM}$ has a direct, linear relationship with $P_{RA}$ above $P_{ref}$.

TGF, on the other hand, responds to variations in sodium concentration at the Macula Densa (MD). The sodium concentration at MD ($\left[ Na^{+} \right]_{MD}$) has a direct relationship with GFR (Section S3.2). We can describe this relationship by a sigmoid function:

| $R_{TGF}=\frac{{(1+K}_{TGF})\times R_{TGF,0}}{1+K_{TGF}\times e^{-\lambda_{TGF}(\left[ Na^{+} \right]_{MD}-\left[ Na^{+} \right]_{MD,0})}}$*,* | (S50) |
| --- | --- |

where $K_{TGF}$ and $\lambda_{TGF}$ are constant coefficients representing the maximum and steepness of the sigmoid function.

The MD cells also promote the release of renin in response to a decrease in $\left[ Na^{+} \right]_{MD}$, which subsequently releases Ang II, and in turn, restricts the efferent arterioles (thereby increasing $R_{Eff}$):

| $R_{Eff}=\frac{K_{Eff}{\times R}_{Eff,0}}{1+\frac{\left[ Ang II \right]_{E,50}}{\left[ Ang II \right]}}$, | (S51) |  |
| --- | --- | --- |
| $\left[ Ang II \right]_{E,50}=\left[ Ang II \right]_{0}(K_{Eff}-1)$, | | (S52) |

where$K_{Eff}$ is a constant coefficient, and $\left[ Ang II \right]_{E,50}$ is the concentration at which $R_{Eff}$ reaches 50% of its maximum value, which is defined by Eq. (S52) to satisfy the initial conditions.

As in our prior work, GFR follows the Starling equation:

| $J_{GFR}=K_{G}\left[ P_{G}-P_{B}-\pi_{G} \right]$, | (S53) |
| --- | --- |

where $K_{G}$ is the glomerular filtration coefficient, $\pi_{G}$the glomerular colloid oncotic pressure, $P_{B}$the hydrostatic pressure of Bowman’s capsule, and $P_{G}$ the glomerular hydrostatic pressure.

To estimate $P_{G}$, we assumed that despite the fluid being filtered across the glomerular capillaries, $P_{G}$ does not change from the inlets to the outlets of the capillaries since arteries pressurize them from both sides [27]. Then, according to Poiseuille's law, the magnitude of the pressure drop from the efferent arterioles to the renal vein is proportional to the flow rate along this path and inversely related to its resistance:

| $P_{G}=P_{RV}+{(R}_{Eff}+R_{RV})(J_{RPF}-UO)\approx P_{RV}+{(R}_{Eff}+R_{RV})(J_{RPF})$. | (S54) |
| --- | --- |

Note that we ignored UO being eliminated from $J_{RPF}$ due to its relative negligibility.

Formulating $\pi_{G}$ is more challenging. The colloid oncotic pressure is a nonlinear function of the albumin concentration in the plasma within the glomerular space. Since albumin stays within the glomerular space while fluid keeps getting filtered into the Bowman’s capsule, there is a steep gradient in $\pi_{G}$, i.e., it increases along the glomerular capillaries. The higher GFR, the steeper the gradient in $\pi_{G}$. But, because GFR itself is a function of $\pi_{G}$, this can cause a chicken and egg problem, calling for a numerical computation method or complex finite element methods to predict how $\pi_{G}$ varies along the tubules [26]. However, we used a different approach. It has been hypothesized and experimentally shown that GFR almost always flows into the Bowman’s capsule until the net Starling forces becomes zero, which is called the equilibrium point [27], [28]. Following Eq. (S53) and considering that both $P_{G}$ and $P_{B}$ remain constant along the nephron tubules, we can write:

| $\pi_{G,f}=P_{G}-P_{B}$, | (S55) |
| --- | --- |

where $\pi_{G,f}$ is the value of $\pi_{G}$ at the equilibrium point, at which the glomerular filtration stops. In addition, since the blood flows freely before it reaches the inlets of the glomerular arterioles, the colloid oncotic pressure at the inlets of the glomerular space equals that of plasma. For simplicity, we assumed that $\pi_{G}$ could be estimated as the average of these critical points:

| $\pi_{G}=\frac{\pi_{G,f}+\pi_{C}}{2}=\frac{P_{G}-P_{B}+\pi_{C}}{2}$*,* | (S56) |
| --- | --- |

where $\pi_{C}$ is the plasma colloid oncotic pressure, given by the mathematical model of VK (Fig. 1).

**S3.2. Reabsorption of Water and Sodium**

See section 2.1.3.2 and Fig. 4 in the manuscript for the description of the general structure of the mathematical model pertaining to water and sodium reabsorption. In the schematic, $J_{XY}$ and $F_{XY}$ represent the flow of water and sodium from node X to node Y, and $J_{XV}$ and $F_{XV}$ represent the water and sodium flow from node X to the renal vein (V), i.e., back to the circulation (reabsorption). At each node, $r_{X}$ and $p_{X}$ represent the fraction of flow reabsorbed into the renal vein pertaining to water and sodium, respectively. The following equations hold for all nodes:

| $J_{XV}=r_{X}J_{(X-1)}$*,* | (S57) |
| --- | --- |
| $J_{XY}={(1-r}_{X})J_{(X-1)}$*,* | (S58) |
| $F_{XV}=p_{X}F_{(X-1)}$*,* | (S59) |
| $F_{XY}={(1-p}_{X})F_{(X-1)}$*,* | (S60) |
| $\left[ Na^{+} \right]_{X}=\frac{F_{XY}}{J_{XY}}$*.* | (S61) |

At the proximal tubules, approximately 65-75% of the filtered water and sodium are reabsorbed together. The glomerulotubular balance ensures that the reabsorption rate is maintained within this range [16], [24], [26]. A second factor that has been shown to affect the proximal tubule reabsorption of sodium (and consequently water due to osmosis), is the activation of angiotensin and aldosterone [29], [30], [31]. We modeled this dependency as a dose-response curve between proximal tubule reabsorption fraction and plasma aldosterone concentration, and bounded the curve from both sides to feasible values, since the proximal tubule reabsorption fraction is mediated by the glomerulotubular balance and cannot astray too far:

$F_{GFR}=J_{GFR}[Na^{+}]$ (S62)

| $r_{P}=p_{P}=0.5+\frac{0.3}{1+\frac{[AL{D]}_{50,P}}{\left[ ALD \right]}}$, | (S63) |
| --- | --- |
| $[AL{D]}_{50,P}=\left[ ALD \right]_{0}(\frac{0.3}{{r_{P}}_{0}-0.5}-1)$, | (S64) |

where $r_{P}$ is the reabsorption fraction at the proximal tubules, $\left[ ALD \right]$ the plasma aldosterone concentration with its nominal value $\left[ ALD \right]_{0}$, and $[AL{D]}_{50,P}$ the plasma aldosterone concentration at which the response reaches 50% of its maximum value (specified so that it satisfies the initial condition for $r_{P}$). Since water and sodium are reabsorbed with the same rate, the sodium concentration doesn’t change at this node and equals that of plasma:

| $\left[ Na^{+} \right]_{P}=\left[ Na^{+} \right]$. | (S65) |
| --- | --- |

The thin descending loop of Henle (LoH) is impermeable to sodium and only water is reabsorbed passively via osmosis, meaning that the longer the fluid is in contact with the surface of nephrons before exiting, the higher the reabsorption fraction in the descending LoH would be. This creates an inverse relationship between water reabsorption fraction and flow rate, which is one of the major players in the phenomenon of pressure diuresis, that is, when BP (and consequently, GFR) increases, water passes the nephrons more quickly and less is reabsorbed, which leads to a disproportionate increase in UO. Assuming that the cross-sectional area ($A_{N}$) and length ($\Delta x_{N}$) of LoH are constant, the time required for water to pass through the descending LoH may be written as follows:

| $\Delta t_{pass}=\frac{\Delta x_{N} [dm]}{V_{N} [\frac{dm}{min}]}=\frac{\Delta x_{N} [dm]}{\frac{J_{PN}\left[ \frac{ml}{min} \right]}{A_{N}\left[ {dm}^{2} \right]}}$*,* | (S66) |
| --- | --- |

where $\Delta t_{pass}$ is the time it takes for the filtrate to pass the descending LoH. Now if we assume that the reabsorption rate is a linear function of $\Delta t_{pass}$, the reabsorption rate may be written as follows:

| $J_{NV}\propto C_{NV}\Delta t_{pass}=\frac{C_{NV}\Delta x_{N} A_{N}}{J_{PN}}$*,* | (S67) |
| --- | --- |
| $r_{N}=\frac{J_{NV}}{J_{PN}}\propto\frac{C_{NV}\Delta x_{N} A_{N}}{J_{PN}^{2}}$*,* | (S68) |

where $r_{N}$ is water reabsorption fraction at node N. This is an indication of the nonlinearity of the relationship between $r_{N}$ and the flow rate. To simplify, we can describe $r_{N}$ as an inverse-sigmoidal function of the flow:

| $r_{N}=\frac{2\times r_{N,0}}{1+e^{\lambda_{N}(\frac{J_{PN}}{J_{PN,0}}-1)}}$*,* | (S69) |
| --- | --- |
| $p_{N}=0$*,* | (S70) |

where $\lambda_{N}$ is a positive constant and $r_{N,0}$ is the nominal value of $r_{N}$. $J_{PN,0}$ and any other nominal flow at a node are calculated by inserting the nominal reabsorption fraction for the node in Eq. (S58). Since only water is reabsorbed, sodium concentration at node N increases relative to node P. According to experimental data from Layton and Layton [32], this increase is about 1.8 times, yielding a value of about 0.44 for $r_{N,0}$ using the following equation:

| $\left[ Na^{+} \right]_{N,0}=\frac{F_{PN,0}}{J_{NK,0}}=\frac{\left[ Na^{+} \right]_{P}}{1-r_{N,0}}=1.8\left[ Na^{+} \right]_{P}$*.* | (S71) |
| --- | --- |

This brings the total reabsorption fraction of water before reaching the collecting duct to approximately 80%, which agrees with Guyton’s findings [33]. We can re-write Eq. (S69) as:

| $r_{N}=\frac{0.88}{1+e^{\lambda_{N}(\frac{J_{PN}}{J_{PN,0}}-1)}}$*.* | (S72) |
| --- | --- |

The thick ascending LoH, on the contrary, is impermeable to water and actively reabsorbs approximately 60% of the remaining sodium. Since our proximal reabsorption fraction is tunable and can take any value between 0.65-0.75, we designed the reabsorption fraction at node K such that at the end of it, collectively 90% of the sodium would be reabsorbed, which is consistent with the literature [33]:

| $r_{K}=0$*,* | (S73) |
| --- | --- |
| $p_{K}=\frac{0.9-r_{P}}{1-r_{P}}$*,* | (S74) |

where $r_{K}$ is the sodium reabsorption fraction at node K. As there is only sodium reabsorption at node K, sodium concentration drastically decreases.

Before reaching MD, the filtrate passes through the outlet of LoH and into the early distal tubules (E). Here, water remains impermeable. In contrast, sodium reabsorption is passive and has an inverse relationship with the flow rate. We used an inverse-sigmoidal function of the flow similar to the passive reabsorption at node N:

| $p_{E}=\frac{1}{1+e^{\lambda_{E} (\frac{F_{KE}}{F_{KE,0}}-1)}}$*,* | (S75) |
| --- | --- |
| $r_{E}=0$*,* | (S76) |

where $\lambda_{E}$ is a constant coefficient and $F_{KE,0}$ is the nominal sodium flow rate from node K to node E. The normal reabsorption fraction at node E is approximately 0.5, which collectively makes sodium reabsorption up to the collecting duct equal to 95% of the glomerular filtration. Hence, sodium concentration at the outlet of node E can be found by dividing sodium flow by water flow at the outlet of the node. Since there is no more reabsorption up to MD:

| $\left[ Na^{+} \right]_{E}=\frac{F_{EC}}{J_{EC}}=\left[ Na^{+} \right]_{MD}$*.* | (S77) |
| --- | --- |

Thus, sodium concentration at MD is determined. Noting that sodium concentration at MD triggers TGF, it has a direct relationship with GFR. Assuming that normal sodium reabsorption at the inlet of MD is 95% of the glomerular sodium flow, the nominal value of $\left[ Na^{+} \right]_{MD}$, an important factor in formulating TGF (see Eq. (S50)), is determined by (subject-specific) total water reabsorption fraction at MD:

| $\left[ Na^{+} \right]_{MD,0}=\frac{F_{EC,0}}{J_{EC,0}}=\frac{\left( 1-0.95 \right)J_{GFR,0}\left[ Na^{+} \right]_{0}}{\left( 1-r_{P_{0}} \right)\left( 1-0.44 \right)J_{GFR,0}}=\frac{0.089\left[ Na^{+} \right]_{0}}{\left( 1-r_{P_{0}} \right)}$*.* | (S78) |
| --- | --- |

The late distal tubule and the collecting ducts are the realm of hormones: the reabsorption of the remaining water is regulated by ADH, while the reabsorption of the remaining sodium is regulated by aldosterone. Specifically, we modeled the reabsorption rates of water and sodium in the late distal tubule and the collecting ducts by the following dose-response curves of ADH concentration in the extracellular fluid $\left[ ADH \right]$, and aldosterone concentration in plasma ($\left[ ALD \right]$), respectively:

| $r_{C}=\frac{r_{C,max}-r_{C,min}}{1+\frac{\left[ ADH \right]_{50}}{\left[ ADH \right]}}+r_{C,min}$*,* | (S79) |
| --- | --- |
| $\left[ ADH \right]_{50}=\left[ ADH \right]_{0}\left( \frac{r_{C,max}-r_{C,min}}{r_{C,0} -r_{C,min}}-1 \right)$*,* | (S80) |
| $p_{C}=\frac{p_{C,max}-p_{C,min}}{1+\left( \frac{\left[ ALD \right]_{50}}{\left[ ALD \right]} \right)^{n_{ALD}}}+p_{C,min}$*,* | (S81) |
| $\left[ ALD \right]_{50}=\left[ ALD \right]_{0}\sqrt[n_{ALD}]{\frac{p_{C,max}-p_{C,min}}{p_{C,0}-p_{C,min}}-1}$*,* | (S82) |
| $J_{UO}=J_{EC}(1-r_{C})$*,* | (S83) |
| $F_{UO}=F_{EC}(1-p_{C})$*,* | (S84) |
| $\left[ Na^{+} \right]_{UO}=\frac{F_{UO}}{J_{UO}}$*,* | (S85) |

where $r_{C}$ is water reabsorption fraction in the collecting ducts mediated by ADH, $p_{C}$ is sodium reabsorption fraction in the collecting ducts mediated by aldosterone, and $n_{ALD}$ is the order of the aldosterone dose-response curve. The subscripts “min” and “max” represent possible minimum and maximum values associated with the reabsorption fractions, which are embedded into the dose-response curves to ensure the reabsorption fraction functions have reasonable upper and lower bounds. $\left[ ADH \right]_{50}$ and $\left[ ALD \right]_{50}$ are plasma ADH and aldosterone concentrations at which their mediated reabsorption fraction reaches 50% of its maximum value. They are determined so that the nominal values for the reabsorption fractions ($r_{C,0}$ and $p_{C,0}$) satisfy Eq. (S79) and Eq. (S81). These last two reabsorption factors play a tremendous role in determining UO and sodium concentration in UO.

While $r_{C,0}$ is optimized in our mathematical model and can take any values within 0.92 to 0.97, the determination of $p_{C,0}$ presents more challenges. The reason is because sodium concentration in UO ($\left[ Na^{+} \right]_{UO}$) can take vastly different values during the day even in a healthy subject (animals or humans), and more importantly, across different species associated with different levels of plasma sodium concentration. Hence, instead of finding the absolute values for $p_{C,0}$, we specify it by setting constraints on the *ratio* ($\alpha_{UO,Na,0})$ of nominal sodium concentration in UO to nominal sodium concentration in PV based on the literature to (i) better individualize the mathematical model as well as to (ii) set plausible values for it. Since we have designed the mathematical model of reabsorption such that normally 95% of the sodium flow is reabsorbed before reaching the collecting ducts, $\alpha_{UO,Na,0}$ can be written as follows:

| $\alpha_{UO,Na,0}=\frac{\left[ Na^{+} \right]_{UO,0}}{\bar{\left[ Na^{+} \right]_{0}}}=\frac{F_{UO,0}/J_{UO,0}}{\bar{\left[ Na^{+} \right]_{0}}}=\frac{J_{GFR}\left( 1-0.95 \right)\left( 1-p_{C,0} \right)\left[ Na^{+} \right]_{0}}{\left[ Na^{+} \right]_{0}J_{GFR}\left( 1-r_{P} \right)\left( 1-0.44 \right)\left( \left( 1-r_{C,0} \right) \right)}.$ | (S86) |
| --- | --- |

Therefore, we can find $p_{C,0}$ as a function of $\alpha_{UO,Na,0}$, $r_{P}$, and $r_{C,0}$using the constraint above:

| $p_{C,0}=1-11.2\times\alpha_{UO,Na}\left( 1-r_{P} \right)\left( 1-r_{C,0} \right).$ | (S87) |
| --- | --- |

**S4. Hormonal System**

**S4.1. Renin-Angiotensin-Aldosterone System**

The renin release rate is assumed to be a linear function of the fractional change in $\left[ Na^{+} \right]_{MD}$:

| $Re^{+}=Re_{0}^{+}(1-\frac{\left[ Na^{+} \right]_{MD}-\left[ Na^{+} \right]_{MD,0}}{\left[ Na^{+} \right]_{MD,0}})$*,* | (S88) |
| --- | --- |

where $Re^{+}$ is renin release rate and $Re_{0}^{+}$ its nominal value. Normally, approximately 60% of renin is metabolized by the blood flow through the liver (hence, determined by CO), 20% cleared by the kidneys (hence, determined by the GFR), and 20% by other tissues, which we assume to remain constant [34]. Considering that initially the excretion rate must equal the release rate to maintain equilibrium, we can write:

| $Re^{-}=\frac{0.6Re_{0}^{+}}{\left[ Re \right]_{0}}\left[ Re \right]\left( 1+\frac{CO-CO_{0}}{CO_{0}} \right)+ \frac{0.2Re_{0}^{+}}{\left[ Re \right]_{0}}\left[ Re \right]\left( 1+\frac{J_{GFR}-J_{GFR,0}}{J_{GFR.0}} \right)+0.2Re_{0}^{+}$, | (S89) |
| --- | --- |
| $\frac{d\left( Re \right)}{dt}=Re^{+}-Re^{-}$*,* | (S90) |
| $\left[ Re \right]=\frac{Re}{V_{P}}$*,* | (S91) |

where $Re^{-}$ is renin metabolism/excretion rate, $Re$ is total renin circulating in the blood, $\left[ Re \right]$ is its plasma concentration, and$\left[ Re \right]_{0}$ is the nominal plasma concentration of renin. Studies have shown that there is a 20–90-minute latency period before Ang II and aldosterone get activated [31], [33]. To simplify the mathematical model, we have incorporated the delay into the renin dynamics, because renin is the precursor to both Ang II and aldosterone:

| $\Delta\left[ Re \right]=\frac{\left[ Re \right]-\left[ Re \right]_{0}}{\left[ Re \right]_{0}}$*,* | (S92) |
| --- | --- |
| $\frac{d\left( \Delta\left[ Re \right]_{del} \right)}{dt}=\frac{\Delta\left[ Re \right]-\Delta\left[ Re \right]_{del}}{\tau_{ren}}$ | (S93) |

where $\Delta\left[ Re \right]$ is the fractional deviation of plasma renin concentration from its nominal value, $\Delta\left[ Re \right]_{del}$ is its delayed effect, and $\tau_{ren}$ is a time constant.

The relationship between Ang II release rate and the delayed renin variation is also linear [16], [35]:

| ${Ang II}^{+}={Ang II}_{0}^{+}(1+C_{Ang}\Delta\left[ Re \right]_{del})$*,* | (S94) |
| --- | --- |

where ${Ang II}^{+}$ is Ang II production rate, ${Ang II}_{0}^{+}$ is its nominal value, and $C_{Ang}$ is a constant coefficient reflecting the sensitivity of Ang II release rate to renin. Since most Ang II is deactivated by its passage through the liver, its rate of metabolism at any instant depends on Ang II concentration and CO. Assuming nominal steady-state conditions, we can write:

| ${Ang II}^{-}=\frac{{Ang II}_{0}^{+}}{\left[ Ang II \right]_{0}}\left[ Ang II \right]\left( 1+\frac{CO-CO_{0}}{CO_{0}} \right)$*,* | (S95) |
| --- | --- |
| $\frac{d\left( Ang II \right)}{dt}={Ang II}^{+}-{Ang II}^{-}$*,* | (S96) |
| $\left[ Ang II \right]=\frac{Ang II}{V_{P}}$*,* | (S97) |

where ${Ang II}^{-}$ is the Ang II metabolism rate.

Aldosterone is primarily secreted in response to an increase in $[Ang II]$ and plasma potassium concentration as well as a decrease in plasma sodium concentration. In our mathematical model, we assumed that plasma potassium concentration is constant, and that aldosterone secretion rate is an exponential function of the weighted sum of the effects of Ang II and plasma sodium concentrations:

| ${ALD}^{+}={ALD}_{0}^{+}e^{(w_{ALD}\Delta{ALD}_{Na}^{+} +(1-w_{ALD})\Delta{ALD}_{Ang}^{+})}$, | (S98) |
| --- | --- |

where ${ALD}^{+}$ is aldosterone secretion rate, ${ALD}_{0}^{+}$ is its nominal value, $w_{ALD}$ is the weight of the effect of $\left[ Na^{+} \right]$, which can take any value between 0 and 1, $\Delta{ALD}_{Na}^{+}$ is the fractional effect of $\left[ Na^{+} \right]$ on aldosterone release rate, and $\Delta{ALD}_{Ang}^{+}$ is the fractional effect of $\left[ Ang II \right]$ on aldosterone release. The relationship between aldosterone secretion and plasma sodium concentration is assumed to be linear:

| $\Delta{ALD}_{Na}^{+}=-C_{ALD,Na}(1-\frac{\left[ Na^{+} \right]-\left[ Na^{+} \right]_{0}}{\left[ Na^{+} \right]_{0}})$*,* | (S99) |
| --- | --- |

where$C_{ALD,Na}$ is a constant. Eq. (S99) suggests that aldosterone concentration increases when $\left[ Na^{+} \right]$ decreases, which results in an increase in the reabsorption of sodium in the collecting ducts to recover normal $\left[ Na^{+} \right]$ levels (Eq. (S81)). The following dose-response curve can capture the relationship between aldosterone release rate and $\left[ Ang II \right]$ based on experiments conducted by Blair et al. [36]:

| $\Delta{ALD}_{Ang}^{+}=\frac{K_{ALD}}{1+\left( \frac{\left[ Ang II \right]_{50}}{\left[ Ang II \right]} \right)^{3}}-1$*,* | (S100) |
| --- | --- |

where $K_{ALD}$ is the maximum fractional increase in $\Delta{ALD}_{Ang}^{+}$, and$\left[ Ang II \right]_{50}$ is the concentration at which $\Delta{ALD}_{Ang}^{+}$ attains 50% of its maximal value. To satisfy nominal steady state condition, $\left[ Ang II \right]_{50}$ is specified as follows:

| $\left[ Ang II \right]_{50}=\left[ Ang II \right]\sqrt[3]{K_{ALD}-1}$*,* | (S101) |
| --- | --- |

Normally, approximately 90% of aldosterone is metabolized in the liver and the remaining 10% is excreted by the kidneys. Hence, aldosterone excretion can be estimated by:

| $ALD^{-}=\frac{0.9{ALD}_{0}^{+}}{\left[ ALD \right]_{0}}\left[ ALD \right]\left( 1+\frac{CO-CO_{0}}{CO_{0}} \right)+ \frac{0.1{ALD}_{0}^{+}}{\left[ ALD \right]_{0}}\left[ ALD \right]\left( 1+\frac{J_{GFR}-J_{GFR,0}}{J_{GFR,0}} \right)$*,* | (S102) |
| --- | --- |
| $\frac{d\left( ALD \right)}{dt}=ALD^{+}-ALD^{-}$*,* | (S103) |
| $\left[ ALD \right]=\frac{ALD}{V_{P}}$*,* | (S104) |

where $ALD^{-}$ is combined metabolism and excretion rates of aldosterone, and $\left[ ALD \right]_{0}$is its nominal concentration in plasma.

**S4.2. Antidiuretic Hormone**

We expressed the dynamics of ADH content by the following phenomenological model:

| $\frac{d\left( ADH \right)}{dt}=K_{ADH}e^{\left( -\lambda_{V_{P}}\Delta V_{P}+\lambda_{\left[ Na^{+} \right]}\Delta\left[ Na^{+} \right] \right)}-0.27K_{ADH}\left[ ADH \right]\frac{J_{GFR}}{J_{GFR,0}}-0.73K_{ADH}\left[ ADH \right]\frac{CO}{CO_{0}}$*,* | (S105) |
| --- | --- |

where $ADH$ is ADH content in the extracellular fluid, $K_{ADH}$ is nominal ADH secretion rate, and $\lambda_{V_{P}}$ and $\lambda_{\left[ Na^{+} \right]}$ are positive constants representing the sensitivity of ADH secretion on the changes in plasma volume ($\Delta V_{P}$) and sodium plasma concentration ($\Delta\left[ Na^{+} \right]$) from their respective nominal values. The coefficients 0.27 and 0.73 in Eq. (S105) originate from the fact that approximately 27% of ADH is excreted by each passage through the glomerulus (as it is not reabsorbed in the kidneys [37]) while the remaining 73% is metabolized in the liver (which is proportional to hepatic blood flow and CO [38]). Noting that ADH concentrations in the extracellular fluid and plasma are equal, and that we use total ADH in the extracellular fluid as a state, we can write:

| $\left[ ADH \right]=\frac{ADH}{V_{P}+V_{BT}+V_{IT}}$*,* | (S106) |
| --- | --- |

where $V_{P}$, $V_{BT}$, and $V_{IT}$ come from the mathematical model of VK.

Table S1: Variable definitions and abbreviations in alphabetical order.

| **Abbreviation** | **Definition** |
| --- | --- |
| ADH | Antidiuretic hormone content in the ECF |
| $[ADH]$ | Plasma (and ECF) antidiuretic hormone concentration |
| $\left[ ADH \right]_{50}$ | Plasma ADH concentration at which $r_{C}$ reaches half its max value |
| ALD | Aldosterone |
| $\left[ ALD \right]$ | Plasma aldosterone concentration |
| $\left[ ALD \right]_{50}$ | Plasma ALD concentration at which $p_{C}$ reaches half its max value |
| $\Delta{ALD}_{Ang}^{+}$ | Fractional effect of $Ang II$ on aldosterone release rate |
| $\Delta{ALD}_{Na}^{+}$ | Fractional effect of $\left[ Na^{+} \right]$ on aldosterone release rate |
| $Ang II$ | Angiotensin $\mathrm{II}$ |
| $\left[ Ang II \right]$ | Angiotensin $\mathrm{II}$ plasma concentration |
| $\left[ Ang II \right]_{50}$ | $\left[ Ang II \right]$ at which $\Delta{ALD}_{Ang}^{+}$ reaches half its max value |
| $ANS$ | Autonomous nervous system |
| $BV$ | Blood volume |
| $BP$ | Blood pressure |
| $CE$ | Cardiac effectiveness |
| *CO* | Cardiac Output |
| $CNS$ | Central nervous system |
| *CV* | Cardiovascular |
| $CVP$ | Central venous pressure |
| $ECF$ | Extracellular fluid |
| $F_{GFR}$ | Rate of glomerular filtration of sodium |
| $F_{I}$ | Rate of sodium flow into ECF by fluid infusion |
| $F_{UO}$ | Rate of sodium excretion from ECF UO |
| $GFR$ | Glomerular filtration rate |
| $J_{GFR}$ | Rate of glomerular filtration of water |
| $J_{I}$ | Rate of water flow into plasma by fluid infusion |
| $J_{RPF}$ | Renal plasma flow rate |
| $J_{UO}$ | Rate of water excretion through UO |
| $LR$ | Lactated Ringer’s solution |
| $LOH$ | Loop of Henle |
| $MAP$ | Mean arterial pressure |
| $MD$ | Macula Densa |
| $M_{MAP}$ | Magnitude of increase in MAP due to post-burn vasoconstriction |
| $MM$ | Myogenic mechanism |
| $MSP$ | Mean systemic pressure |
| $[Na^{+}]$ | Plasma sodium concentration |
| $\left[ Na^{+} \right]_{E}$ | Sodium concentration at the early distal tubules |
| $\left[ Na^{+} \right]_{MD}$ | Sodium concentration at the Macula Densa |
| $\left[ Na^{+} \right]_{N}$ | Sodium concentration at the thin descending LOH |
| $\left[ Na^{+} \right]_{P}$ | Sodium concentration at the proximal tubules |
| $\left[ Na^{+} \right]_{UO}$ | Sodium concentration in UO |
| $p_{E}$ | Water reabsorption fraction at early distal tubules |
| $p_{C}$ | Sodium reabsorption fraction at the collecting ducts |
| $P_{G}$ | Glomerular hydrostatic pressure |
| $p_{K}$ | Water reabsorption fraction at the tick ascending LOH |
| $p_{N}$ | Sodium reabsorption fraction at the thin descending LOH |
| $p_{P}$ | Sodium reabsorption fraction at the proximal tubules |
| $P_{RA}$ | Renal arterial pressure |
| $P_{RV}$ | Renal vein pressure |
| $\pi_{C}$ | Capillary colloid oncotic pressure |
| $\pi_{G}$ | Glomerular colloid oncotic pressure |
| $RAAS$ | Renin-angiotensin-aldosterone system |
| $R_{Aff}$ | Renal afferent resistance |
| $RAP$ | Right atrial pressure |
| $r_{C}$ | Water reabsorption fraction at the collecting ducts |
| $Re$ | Renin content in blood |
| $r_{E}$ | Water reabsorption fraction at early distal tubule |
| $\Delta\left[ Re \right]$ | Fractional deviation of plasma renin concentration from baseline |
| $\Delta\left[ Re \right]_{del}$ | Delayed $\Delta\left[ Re \right]$ |
| $R_{Eff}$ | Renal efferent resistance |
| $r_{K}$ | Water reabsorption fraction at the tick ascending LOH |
| $R_{MM}$ | Afferent resistance contributing to myogenic mechanism |
| $r_{N}$ | Water reabsorption fraction at the thin descending LOH |
| $r_{Na}$ | Total sodium reabsorption fraction |
| $r_{P}$ | Water reabsorption fraction at the proximal tubules |
| $RPF$ | Renal plasma flow |
| $R_{RV}$ | Renal vein resistance |
| $R_{TGF}$ | Afferent resistance contributing to Tubuglomerular Feedback |
| $RVR$ | Return venous resistance |
| $r_{W}$ | Total water reabsorption fraction |
| $SS$ | Subject-specific |
| $TBSA$ | Total burned surface area |
| $TGF$ | Tubuglomerular Feedback |
| $TPR$ | Total peripheral resistance |
| $UO$ | Urinary output |
| $V_{BT}$ | Burn tissue volume |
| $V_{IT}$ | Intact tissue volume |
| $VK$ | Volume kinetics |
| $V_{P}$ | Plasma volume |
| $VR$ | Venous return |
| $W$ | Weight |

Table S2: Parameter values are reported as median (IQR). Categories: “L”: subject-invariant parameters taken from **l**iterature. “D”: values taken from the measurements in the **d**ataset, “S”: **s**ubject-specific parameters inferred by fitting the model to data of a subject, “P”: subject-invariant parameters inferred by fitting the model to data of **p**opulation. “-”: parameters determined from model constraints. The references provided for L parameters are the source of the parameter values. The references provided for P and S parameters are the sources used to verify the physiological plausibility. For all the parameters, the subscript “0” indicates the nominal values.

| Symbol | Pigs | | Sheep | | Humans | |
| --- | --- | --- | --- | --- | --- | --- |
|  | Category | Value | Category | Value | Category | Value |
| 1. **Volume Kinetics Parameters** | | | | | | |
| $BV_{0}$ [ml/kg] | L | 60 | L | 65 [39] | L | 83 [3], [40] |
| $HCT_{0}$ [.] | D | 0.28 (0.) | D | 0.30 (0.056) | L | 0.45 |
| $V_{P,0}$ [ml/kg] | L | $BV_{0}(1-HCT_{0})$ | L | $BV_{0}(1-HCT_{0})$ | L | 45.7 [3], [40] |
| $\varepsilon_{B}$ [.] | D | 0.3-0.4 | D | 0.4 | D | 0.2-0.98 |
| $S_{B}$[m^2^] | L | 1 | L | 1 [41] | D | 2.08 (0.44) [1] |
| $\bar{V}_{BT}$ [ml/kg] | - | 120$\varepsilon_{B}r_{FV}$ | | | | |
| $\bar{V}_{IT}$ [ml/kg] | - | 120$\left( 1-\varepsilon_{B}r_{FV} \right)$ | | | | |
| $r_{FV}$ [.] | L | 0.28 [3] | L | 0.28 [3] | L | 0.28 [3] |
| $\left[ A \right]_{P,0}$ [g/ml] | L | 0.059 [42] | L | 0.059 [43] | L | 0.045 [44] |
| $\left[ A \right]_{BT,0}$ [g/ml] | L | 0.028 [42][43] | L | 0.028 [42] | L | 0.018 [3], [45] |
| $\left[ A \right]_{IT,0}$ [g/ml] | L | 0.028 [42] | L | 0.028 [42] | L | 0.018 [3], [45] |
| $\bar{A}_{P}$ [g] | - | $[\bar{A}_{P}]\bar{V}_{P}$ | | | | |
| $\bar{A}_{BT}$ [g] | - | $[\bar{A}_{BT}]\bar{V}_{BT}$ | | | | |
| $\bar{A}_{IT}$ [g] | - | ${[\bar{A}}_{IT}$]$\bar{V}_{IT}$ | | | | |
| $J_{C,0}$ [ml/h] | S | 45.47 (27.99) | S | 81.94 (18.6) | S | 139.82 (64.81) [46] |
| $J_{L,0}$ [ml/h] | - | ${J_{C,0} -K}_{1,EV}S_{B}$ [3] | | | | |
| $\bar{J}_{L,BT}$ [ml/h] | - | $J_{L,0}\varepsilon_{B}r_{FV}$ | | | | |
| $\bar{J}_{L,IT}$ [ml/h] | - | $J_{L,0}\left( 1-\varepsilon_{B}r_{FV} \right)$ | | | | |
| $C_{O}$ [mmHg/g/ml] | L | 250 [42] | L | 250 [42] | L | 609 [3] |
| $C_{L}$ [.] | S | 0.19 (0.33) | S | 0.28 (0.19) | P | 0.054 |
| $S_{L}$ [1/mmHg] | S | 6.94 (1.51) | S | 6.67 (1.96) | S | 1.15 (0.74) |
| $P_{C,0}$ [mmHg] | S | 7.61 (1.33) [43] | S | 7.00 (0.71) | P | 16.63 [47], [48] |
| $E_{C}$ [mmHg/ml] | S | 0.0187 (0.0078) | S | 0.0162 (0.0036) | P | 0.008 [49] |
| $\alpha$ [mmHg] | L | 10 [5] | L | 10 [5] | L | 10 [5] |
| $\gamma$[mmHg] | L | 3.75 [5] | L | 3.75 [5] | L | 3.75 [5] |
| $\hat{y}$ | L | 4 [5] | L | 4 [5] | L | 4 [5] |
| $\check{y}$ | L | 1 [5] | L | 1 [5] | L | 1 [5] |
| $\hat{R}$ | L | 3.5 [5] | L | 3.5 [5] | L | 3.5 [5] |
| $\hat{R}$ | L | 0.23 [5] | L | 0.23 [5] | L | 0.23 [5] |
| $n$ | L | 8 [5] | L | 8 [5] | L | 8 [5] |
| $\bar{W}_{X}$ | L | 0.66 [5] | L | 0.66 [5] | L | 0.66 [5] |
| $\hat{Q}_{PD}$ [g/h] | S | 83.93 (98.84) | S | 93.18 (80.82) | P | 99.23 |
| $\lambda_{PD}$ [1/h] | L | 10 [3] | L | 10 [3] | L | 10 [3] |
| ${M_{P}}_{BT}$ [mmHg] | S | 50.28 (43.71) [50] | S | 15.23 (43) [50] | P | 51.63 [50] |
| $\lambda_{1,P_{BT}}$ [1/h] | S | 6.28 (4.02) | S | 6.05 (3.27) | P | 5.62 |
| $\mu$ [.] | S | 562.99 (462.83) | S | 332.83 (274.87) | P | 340.30 |
| $\lambda_{2,P_{BT}}$ [1/h] | - | 8$\lambda_{1,P_{BT}}$ | | | | |
| $K_{1,EV}$ [ml/h/m^2^] | L | 18.48 [9] | L | 18.48 | L | 18.48 |
| $\lambda_{1,EV}$ [1/h] | L | 0.073 | L | 0.073 | L | 0.073 |
| $K_{2,EV}$ [ml/h/m^2^] | L | 28.68 | L | 28.68 | L | 28.68 |
| $\lambda_{2,EV}$ [1/h] | L | -0.0052 | L | -0.0052 | L | -0.0052 |
| $K_{EX}$ [ml/h/m^2^] | L | 25 [44] | L | 25 [44] | L | 25 [8] |
| $\lambda_{EX}$ [1/h] | L | -0.0038 [8] | L | -0.0038 [62] | L | -0.0038 [8] |
| $\eta_{EX}$ [.] | S | 0.63 (0.22) | S | 0.54 (0.38) | P | 0.68 |
| $\bar{\alpha}$ [.] | S | 0.83 (0.14) | S | 0.86 (0.05) | S | 0.87 (0.08) [49] |
| ${1-k}_{PD,BT}$ [.] | S | 0.42 (0.32) | S | 0.36 (0.35) | P | 0.45 [51] |
| $M_{\alpha_{BT}}$ [.] | S | 0.34 (0.38) | S | 0.24 (0.30) | P | 0.32 [50] |
| $M_{\alpha_{IT}}$ [.] | S | 0.12 (0.13) [52] | S | 0.17 (0.08) [52] | S | 0.27 (0.11) [52] |
| $\lambda_{1,\alpha}$ [1/h] | S | 0.04 (0.02) [3] | S | 0.017 (0.024) | P | 0.018 [3] |
| $\lambda_{2,\alpha}$ [1/h] | - | $\mu\lambda_{1,\alpha}$ | | | | |
| $M_{P_{C}}$ [mmHg] | S | 15.41 (27.93) [53] | S | 12.59 (10.30) [53] | S | 21.54 (38) [53] |
| $\lambda_{1,P_{C}}$ [1/h] | S | 0.34 (0.64) | S | 0.62 (0.37) | P | 0.61 |
| $\lambda_{2,P_{C}}$ [1/h] | - | $\mu\lambda_{1,P_{C}}$ | | | | |
| 1. **Cardiovascular Model Parameters** | | | | | | |
| $\lambda_{msp}$ [1/ml] | S | 0.91 (0.31) | S | 1.05 (0.56) | S | 0.36 (0.51) |
| $K_{TPR}$ [.] | S | 4.75 (5.15) | S | 1.50 (3.71) | S | 4.51 (7.58) |
| $\lambda_{TPR}$ [ml/ng] | S | 8.68 (5.97) | S | 6.61 (11.41) | P | 15.57 |
| $\left[ Ang II \right]_{0}$ [ng/ml] | L | 0.027 [16] | L | 0.027 [16] | L | 0.027 [16] |
| $M_{CO}$ [.] | S | 2.28 (1.03) | S | 3.27 (1.45) | P | 2.87 |
| $MAP_{0}$ [mmHg] | S | 66.55 (14.13) | D | 93 (9.25) | P | 84.15 [24] |
| ${CO}_{0}$ [ml/min/kg] | S | 74.64 (32.77) [67] | D | 121 (33.75) | P | 78.72 |
| $M_{MAP}$ [mmHg] | S | 35.54 (41.55) | S | 69.14 (27.917) | P | 45.85 |
| $\lambda_{1MAP}$ [1/h] | S | 0.65 (0.36) | S | 0.51 (0.71) | P | 0.52 |
| $\lambda_{2,MAP}$ [1/h] | S | 341.43 (288.44) | S | 376.21 (333.97) | P | 314.08 |
| $CVP_{0}$ [mmHg] | S | 5.42 (3.56) [42] | S | 2.17 (2.68) | P | 4.34 |
| 1. **Renal Function Model Parameters** | | | | | | |
| $K_{RA}$ [.] | S | 0.92 (0.70) | S | 0.85 (0.46) | P | 1.02 |
| $K_{RV}$ [.] | S | 0.50 (0.28) | S | 0.76 (0.44) | P | 0.98 |
| $R_{A,0}$[mmHg.min/ml] | L | 1.76/W [24] | L | 1.76/W [24] | L | 1.76/W [24] |
| $P_{ref}$ [mmHg] | L | 74 [24] | L | 74 [24] | L | 74 [24] |
| $G_{MM}$ [.] | L | 0.0867 | L | 0.0867 | L | 0.0867 |
| $K_{TGF}$ [.] | S | 2.96 (1.09) | S | 2.78 (1.05) | P | 2.56 |
| $\lambda_{TGF}$ [ml/mEq] | S | 18.40 (13.54) | S | 17.54 (8.78) | P | 18.09 |
| $R_{TGF,0}$ [mmHg.min/ml] | L | 0.4750/W [24] | L | 0.4750/W [24] | L | 0.4750/W [24] |
| $R_{Eff,0}$ [mmHg.min/ml] | L | 4.81/W [24] | L | 4.81/W [24] | L | 4.81/W [24] |
| $K_{Eff}$ [.] | S | 0.13 (0.08) | S | 0.19 (0.52) | S | 0.42 (0.81) |
| $K_{G}$ [ml/min/mmHg] | S | 4.14 (1.12) [54] | S | 3.10 (1.96) [54] | P | 5.14 [54] |
| $P_{B}$ [mmHg] | L | 18 [55] | L | 18 [55] | L | 15 [55] |
| $J_{RPF,0}$ [ml/min] | L | 8.93W [23] | L | 8.93W [23] | L | 8.93W [23] |
| $\left[ ALD \right]_{0}$ [ng/ml] | L | 0.085 [16] | L | 0.085 [16] | L | 0.085 [16] |
| ${r_{P}}_{0}$ [.] | S | 0.67(0.04) [16] | S | 0.72 (0.03) [16] | P | 0.69 [16] |
| $\lambda_{N}$ [.] | S | 0.24 (1.35) | S | 1.77 (1.43) | P | 0.89 |
| $\lambda_{E}$ [.] | S | 4.72 (4.25) | S | 3.03 (5.52) | P | 5.58 |
| $p_{E,0}$ [.] | L | 0.5 | L | 0.5 | L | 0.5 |
| $\left[ Na^{+} \right]_{0}$ [mEq/ml] | D | 139 (2.75) | L | 0.144 | L | 0.144 |
| $r_{C,max}$ [.] | L | 0.995 | L | 0.995 | L | 0.995 |
| $r_{C,min}$ [.] | L | 0.45 | L | 0.45 | L | 0.45 |
| $r_{C,0}$ [.] | S | 0.93 (0.02) | S | 0.94 (0.02) | P | 0.94 |
| $\left[ ADH \right]_{0}$ [pg/ml] | L | 1 [56] | L | 1 [56] | L | 1 [56] |
| $p_{C,max}$ [.] | L | 1 | L | 1 | L | 1 |
| $p_{C,min}$ [.] | L | 0.2 | L | 0.2 | L | 0.2 |
| $n_{ALD}$ [.] | L | 1 | L | 1 | L | 1 |
| $\alpha_{UO,Na,0}$ [.] | S | 0.66 (0.30) | S | 0.38 (0.11) | P | 0.53 |
| 1. **Hormonal System Model Parameters** | | | | | | |
| $Re_{0}^{+}$ [GBU/h] | L | 0.48 [16] | L | 0.48 [16] | L | 0.48 [16] |
| $Re_{0}$ | - | $\left[ Re \right]_{0}V_{PL,0}$ | | | | |
| $\left[ Re \right]_{0}$ [GBU/ml] | L | 0.00006 | L | 0.00006 | L | 0.00006 |
| $\tau_{ren}$ [1/h] | S | 0.53 (0.31) [30] | S | 0.44 (0.42) [30] | P | 0.42 [30] |
| ${Ang II}_{0}^{+}$ [ng/h] | L | 6300 [16] | L | 6300 [16] | L | 6300 [16] |
| $C_{Ang}$ [.] | S | 1.42 (1.64) | S | 0.50 (1.04) | S | 0.88 (1.45) |
| ${ALD}_{0}^{+}$ [ng/h] | L | 3162 [16] | L | 3162 [16] | L | 3162 [16] |
| $w_{ALD}$ [.] | S | 0.69 (0.82) | S | 0.93 (0.21) | P | 0.58 |
| $C_{ALD,Na}$ [.] | S | 23.47 (31.42) | S | 27.52 (39.06) | P | 38.54 |
| $K_{ALD}$ [.] | S | 1.99 (2.07) | S | 1.77 (1.46) | P | 2.67 |
| $K_{ADH}$ [pg/kg·h] | L | 287[37], [38] | L | 287 [37], [38] | L | 287 [37], [38] |
| $\lambda_{V_{P}}$ [1/ml] | S | 0.0009 (0.0048) | S | 0.0003 (0.0009) | S | 0.0007 (0.0009) |
| $\lambda_{\left[ Na^{+} \right]}$ [1/mEq] | S | 37.63 (169.23) | S | 76.12 (105.63) | P | 70.62 |

**References**

[1] G. Arabidarrehdor, A. Tivay, C. Meador, G. C. Kramer, J.-O. Hahn, and J. Salinas, “Mathematical Modeling, In-Human Evaluation, and Analysis of Volume Kinetics and Kidney Function after Burn Injury and Resuscitation,” *IEEE Trans Biomed Eng*, pp. 1–1, 2021, doi: 10.1109/TBME.2021.3094515.

[2] G. Arabidarrehdor *et al.*, “Mathematical model of volume kinetics and renal function after burn injury and resuscitation,” *Burns*, vol. 47, no. 2, pp. 371–386, Mar. 2021, doi: 10.1016/J.BURNS.2020.07.003.

[3] R. T. Ampratwum, B. D. Bowen, T. Lund, R. K. Reed, and J. L. Bert, “A model of fluid resuscitation following burn injury: formulation and parameter estimation,” *Comput Methods Programs Biomed*, vol. 47, no. 1, pp. 1–19, 1995.

[4] E. H. Bresler and L. J. Groome, “On equations for combined convective and diffusive transport of neutral solute across porous membranes.,” *Am J Physiol*, vol. 241, no. 5, pp. F469-76, Nov. 1981, doi: 10.1152/ajprenal.1981.241.5.F469.

[5] A. H. Øien and H. Wiig, “Modeling In Vivo Interstitial Hydration-Pressure Relationships in Skin and Skeletal Muscle,” *Biophys J*, vol. 115, no. 5, pp. 924–935, 2018, doi: 10.1016/j.bpj.2018.07.025.

[6] F.-R. Curry, C. Michel, E. Renkin, and S. Geiger, “Mechanics and thermodynamics of transcapillary exchange,” Jan. 01, 1984.

[7] M. Jarzynska and M. Pietruszka, “The application of the Kedem-Katchalsky equations to membrane transport of ethyl alcohol and glucose,” *Desalination*, vol. 280, no. 1–3, pp. 14–19, 2011, doi: 10.1016/j.desal.2011.07.034.

[8] J. Baudoin, P. Jafari, J. Meuli, L. A. Applegate, and W. Raffoul, “Topical negative pressure on burns: An innovative method for wound exudate collection,” *Plast Reconstr Surg Glob Open*, vol. 4, no. 11, 2016, doi: 10.1097/GOX.0000000000001117.

[9] R. Xiang. Xu, Xia. Sun, and B. S. Weeks, *Burns regenerative medicine and therapy*. Karger, 2004.

[10] A. C. Guyton, “Effect of blood volume, mean circulatory filling pressure, cardiac output, and autoregulation on arterial pressure,” in *Circulatory Physiology III Arterial Pressure and Hypertension*, Saunders W B Co, 1982, ch. 5, pp. 71–86.

[11] R. W. Lee, L. D. Lancaster, R. G. Gay, M. Paquin, and S. Goldman, “Use of acetylcholine to measure total vascular pressure-volume relationship in dogs,” *https://doi.org/10.1152/ajpheart.1988.254.1.H115*, vol. 254, no. 1, 1988, doi: 10.1152/AJPHEART.1988.254.1.H115.

[12] T. E. Lohmeier *et al.*, “Influence of Prolonged Baroreflex Activation on Arterial Pressure in Angiotensin Hypertension,” *Hypertension*, vol. 46, no. 5, pp. 1194–1200, Nov. 2005, doi: 10.1161/01.HYP.0000187011.44201.2e.

[13] A. W. COWLEY, J. F. LIARD, and A. C. GUYTON, “Role of the Baroreceptor Reflex in Daily Control of Arterial Blood Pressure and Other Variables in Dogs,” *Circ Res*, vol. 32, no. 5, pp. 564–576, May 1973, doi: 10.1161/01.RES.32.5.564.

[14] O. W, B. JJ, F. R, L. AF, M. JJ, and R. JI, “Sensitization of the adrenal cortex to angiotensin II in sodium-deplete man,” *Circ Res*, vol. 34, no. 1, pp. 69–77, Apr. 1974, doi: 10.1161/01.RES.40.4.69.

[15] A. C. Guyton, A. W. Lindsey, B. Abernathy, T. Richardson, and uf Ptbyriulogy, “Venous Return at Various Right Atria1 Pressures and the Normal Venous Return Curve’ School of &fed ihe, ABSTRACT”.

[16] R. J. Uttamsingh, M. S. Leaning, J. A. Bushman, E. R. Carson, and L. Finkelstein, “Mathematical model of the human renal system,” *Med Biol Eng Comput*, vol. 23, no. 6, pp. 525–535, 1985, doi: 10.1007/BF02455306.

[17] K. Sunagawa, “Guyton’s venous return curves should be taught at medical schools (complete English translation of Japanese version),” *J Physiol Sci*, vol. 67, no. 4, pp. 447–458, Jul. 2017, doi: 10.1007/S12576-017-0533-0.

[18] A. C. GUYTON, “Determination of cardiac output by equating venous return curves with cardiac response curves,” *Physiol Rev*, vol. 35, no. 1, pp. 123–129, Jan. 1955, doi: 10.1152/PHYSREV.1955.35.1.123.

[19] A. C. GUYTON, A. W. LINDSEY, B. ABERNATHY, and J. B. LANGSTON, “Mechanism of the increased venous return and cardiac output caused by epinephrine,” *Am J Physiol*, vol. 192, no. 1, pp. 126–130, Jan. 1958, doi: 10.1152/AJPLEGACY.1957.192.1.126.

[20] Y. A. Kara and Y. A. Kara, “Burn Etiology and Pathogenesis,” *Hot Topics in Burn Injuries*, May 2018, doi: 10.5772/INTECHOPEN.71379.

[21] S. Soussi, F. Dépret, M. Benyamina, and M. Legrand, “Early hemodynamic management of critically Ill burn patients,” *Anesthesiology*, vol. 129, no. 3, pp. 583–589, 2018, doi: 10.1097/ALN.0000000000002314.

[22] F. R. E. Curry and R. H. Adamson, “Vascular permeability modulation at the cell, microvessel, or whole organ level: towards closing gaps in our knowledge,” *Cardiovasc Res*, vol. 87, no. 2, pp. 218–229, Jul. 2010, doi: 10.1093/CVR/CVQ115.

[23] J. Hall and A. Guyton, “Urine Formation by the Kidneys: I. Glomerular Filtration, Renal Blood Flow, and Their Control,” in *Textbook of Medical Physiology*, ch. Chapter 26.

[24] B. J. Czerwin, S. Patel, C. M. Chiofolo, J. Yuan, and N. W. Chbat, “Modeling the Steady-State Effects of Mean Arterial Pressure on the Kidneys,” *IEEE Open J Eng Med Biol*, vol. 2, 2021, doi: 10.1109/OJEMB.2020.3036547.

[25] L. C. Moore, A. Rich, and D. Casellas, “Ascending myogenic autoregulation: interactions between tubuloglomerular feedback and myogenic mechanisms,” *Bull Math Biol*, vol. 56, no. 3, pp. 391–410, May 1994, doi: 10.1007/BF02460464.

[26] R. Moss and S. R. Thomas, “Hormonal regulation of salt and water excretion: A mathematical model of whole kidney function and pressure natriuresis,” *Am J Physiol Renal Physiol*, vol. 306, no. 2, pp. 183–185, 2014, doi: 10.1152/ajprenal.00089.2013.

[27] B. M. Brenner, J. L. Troy, T. M. Daugharty, W. M. Deen, and C. R. Robertson, “Dynamics of glomerular ultrafiltration in the rat. II. Plasma-flow dependence of GFR,” *https://doi.org/10.1152/ajplegacy.1972.223.5.1184*, vol. 223, no. 5, pp. 1184–1190, 1972, doi: 10.1152/AJPLEGACY.1972.223.5.1184.

[28] W. M. Deen, C. R. Robertson, and B. M. Brenner, “A model of glomerular ultrafiltration in the rat,” *Am J Physiol*, vol. 223, no. 5, pp. 1178–1183, 1972, doi: 10.1152/AJPLEGACY.1972.223.5.1178.

[29] D. W. Good, “Nongenomic actions of aldosterone on the renal tubule,” *Hypertension*, vol. 49, no. 4, pp. 728–739, Apr. 2007, doi: 10.1161/01.HYP.0000259797.48382.b2.

[30] S. A. Salyer *et al.*, “Aldosterone regulates Na+, K+ ATPase activity in human renal proximal tubule cells through mineralocorticoid receptor,” *Biochimica et Biophysica Acta (BBA) - Molecular Cell Research*, vol. 1833, no. 10, pp. 2143–2152, Oct. 2013, doi: 10.1016/J.BBAMCR.2013.05.009.

[31] A. C. Guyton, “Role of Aldosterone in Fluid Volume and Electrolyte Control,” in *Dynamics and Control of the Body Fluids, Circulatory Physiology II*, W.B. Saunders Company, 1975, ch. 20, p. 291.

[32] A. T. Layton and H. E. Layton, “A computational model of epithelial solute and water transport along a human nephron,” *PLoS Comput Biol*, vol. 15, no. 2, Feb. 2019, doi: 10.1371/JOURNAL.PCBI.1006108.

[33] Guyton and Hall, “Urine Formation by the Kidneys: II. Tubular Reabsorption and Secretion,” in *Textbook of Medical Physiology*.

[34] J. Bohlender, J. Ménard, D. Ganten, and F. C. Luft, “Angiotensinogen Concentrations and Renin Clearance,” *Hypertension*, vol. 35, no. 3, pp. 780–786, 2000, doi: 10.1161/01.HYP.35.3.780.

[35] E. G. Schneider, H. H. Rostorfer, and F. D. Nash, “Distribution volume and metabolic clearance rate of renin in anesthetized nephrectomized dogs,” *https://doi.org/10.1152/ajplegacy.1968.215.5.1115*, vol. 215, no. 5, pp. 1115–1122, 1968, doi: 10.1152/AJPLEGACY.1968.215.5.1115.

[36] J. R. BLAIR-WEST *et al.*, “Humoral stimulation of adrenal cortical secretion,” *J Clin Invest*, vol. 41, no. 8, pp. 1606–1627, 1962, doi: 10.1172/JCI104619.

[37] G. Baumann and J. F. Dingman, “Distribution, blood transport, and degradation of antidiuretic hormone in man,” *Journal of Clinical Investigation*, vol. 57, no. 5, pp. 1109–1116, 1976, doi: 10.1172/JCI108377.

[38] H. HELLER and S. M. ZAIDI, “The metabolism of exogenous and endogenous antidiuretic hormone in the kidney and liver in vivo.,” *Br J Pharmacol Chemother*, vol. 12, no. 3, pp. 284–292, 1957, doi: 10.1111/j.1476-5381.1957.tb00136.x.

[39] D. J. Gillett and D. F. J. Halmagyi, “Results and limitations of blood volume measurements in sheep,” *Journal of Surgical Research*, vol. 6, no. 5, pp. 211–214, May 1966, doi: 10.1016/S0022-4804(66)80018-5.

[40] J. P. Coghlan, J. S. Fan, B. A. Scoggins, and A. A. Shulkes, “Measurement of extracellular fluid volume and blood volume in sheep.,” *Aust J Biol Sci*, vol. 30, no. 1–2, pp. 71–84, Apr. 1977, Accessed: Feb. 19, 2020. [Online]. Available: http://www.ncbi.nlm.nih.gov/pubmed/901309

[41] J. W. Bennett, “Regional body surface area of sheep,” *J. agric. Sci., Camb*, vol. 81, pp. 429–432, 2020, doi: 10.1017/S0021859600086469.

[42] G. C. Kramer, R. A. Gunther, M. L. Nerlich, S. S. Zweifach, and R. H. Demling, “Effect of dextran-70 on increased microvascular fluid and protein flux after thermal injury.,” *Circ Shock*, vol. 9, no. 5, pp. 529–41, 1982.

[43] G. C. Kramer, B. A. Harms, R. A. Gunther, E. M. Renkin, and R. H. Demling, “The Effects of Hypoproteinemia on Blood-to-Lymph Fluid Transport in Sheep Lung,” 1981.

[44] J. D. Hoppe, P. C. Scriba, and H. Klüter, “Transfusion Medicine and Hemotherapy - Chapter 5. Human Albumin,” in *Transfusion medicine and hemotherapy : offizielles Organ der Deutschen Gesellschaft fur Transfusionsmedizin und Immunhamatologie*, vol. 36, no. 6, Karger Publishers, 2009, ch. 5 Human Al, pp. 399–407.

[45] M. Ellmerer *et al.*, “Measurement of interstitial albumin in human skeletal muscle and adipose tissue by open-flow microperfusion,” *Am J Physiol Endocrinol Metab*, vol. 278, no. 2 41-2, 2000, doi: 10.1152/ajpendo.2000.278.2.e352.

[46] S. L. Xie, R. K. Reed, B. D. Bowen, and J. L. Bert, “A Model of Human Microvascular Exchange,” 1995. doi: 10.1006/mvre.1995.1012.

[47] R. H. Demling, “The Burn Edema Process : Current Concepts,” *Journal of Burn Care & Rehabilitation*, vol. 26, pp. 207–227, 2005, doi: 10.1097/01.BCR.0000162151.71482.B3.

[48] J. Hall and A. Guyton, “Cardiac Failure,” in *Textbook of Medical Physiology*, ch. 22, Cardia, p. 276.

[49] C. Chapple, B. D. Bowen, R. K. Reed, S. L. Xie, and J. L. Bert, “A model of human microvascular exchange: parameter estimation based on normals and nephrotics,” *Comput Methods Programs Biomed*, vol. 41, no. 1, pp. 33–54, 1993, doi: 10.1016/0169-2607(93)90064-R.

[50] T. Lund, H. Onarheim, H. Wiig, and R. K. Reed, “Mechanisms behind increased dermal imbibition pressure in acute burn edema,” *Am J Physiol Heart Circ Physiol*, vol. 256, no. 4, 1989, doi: 10.1152/ajpheart.1989.256.4.h940.

[51] J. L. Bert, B. D. Bowen, R. K. Reed, and H. Onarheim, “Microvascular exchange during burn injury: IV. Fluid resuscitation model.,” *Circ Shock*, vol. 34, no. 3, pp. 285–97, Jul. 1991.

[52] L. Kongstad, A. D. Möller, and P. O. Grände, “Reflection coefficient for albumin and capillary fluid permeability in cat calf muscle after traumatic injury,” *Acta Physiol Scand*, vol. 165, no. 4, pp. 369–377, Apr. 1999, doi: 10.1046/j.1365-201x.1999.00521.x.

[53] R. M. Pitt, J. C. Parker, G. J. Jurkovich, A. E. Taylor, and P. W. Curreri, “Analysis of altered capillary pressure and permeability after thermal injury,” *Journal of Surgical Research*, vol. 42, no. 6, pp. 693–702, 1987, doi: 10.1016/0022-4804(87)90013-8.

[54] M. Nesje, A. Flåøyen, and L. Moe, “Estimation of glomerular filtration rate in normal sheep by the disappearance of iohexol from serum,” *Vet Res Commun*, vol. 21, no. 1, pp. 29–35, 1997, doi: 10.1023/B:VERC.0000009698.28252.d1.

[55] Guyton and Hall, “The Urinary System: Functional Anatomy and Urine Formation by the Kidneys,” in *Medical Physiology*, ch. 26.

[56] F. M. Toates and K. Oatley, “Computer simulation of thirst and water balance,” *Med Biol Eng*, vol. 8, no. 1, pp. 71–87, 1970, doi: 10.1007/BF02551751.
